# B cell profiles, antibody repertoire and reactivity reveal dysregulated responses with autoimmune features in melanoma

**DOI:** 10.1038/s41467-023-39042-y

**Published:** 2023-06-08

**Authors:** Silvia Crescioli, Isabel Correa, Joseph Ng, Zena N. Willsmore, Roman Laddach, Alicia Chenoweth, Jitesh Chauhan, Ashley Di Meo, Alexander Stewart, Eleni Kalliolia, Elena Alberts, Rebecca Adams, Robert J. Harris, Silvia Mele, Giulia Pellizzari, Anna B. M. Black, Heather J. Bax, Anthony Cheung, Mano Nakamura, Ricarda M. Hoffmann, Manuela Terranova-Barberio, Niwa Ali, Ihor Batruch, Antoninus Soosaipillai, Ioannis Prassas, Antigona Ulndreaj, Miyo K. Chatanaka, Rosamund Nuamah, Shichina Kannambath, Pawan Dhami, Jenny L. C. Geh, Alastair D. MacKenzie Ross, Ciaran Healy, Anita Grigoriadis, David Kipling, Panagiotis Karagiannis, Deborah K. Dunn-Walters, Eleftherios P. Diamandis, Sophia Tsoka, James Spicer, Katie E. Lacy, Franca Fraternali, Sophia N. Karagiannis

**Affiliations:** 1 St John’s Institute of Dermatology, School of Basic & Medical Biosciences, King’s College London, Guy’s Hospital, London, UK; 2 Randall Centre for Cell and Molecular Biophysics, King’s College London, London, UK; 3 Research Department of Structural and Molecular Biology, University College London, London, UK; 4 Department of Informatics, Faculty of Natural, Mathematical and Engineering Sciences, King’s College London, London, UK; 5 Breast Cancer Now Research Unit, School of Cancer & Pharmaceutical Sciences, King’s College London, Guy’s Hospital, London, UK; 6 Lunenfeld-Tanenbaum Research Institute, Mount Sinai Hospital, Toronto, ON Canada; 7 School of Biosciences and Medicine, University of Surrey, Guildford, UK; 8 Peter Gorer Department of Immunobiology, School of Immunology and Microbial Sciences, Faculty of Life Sciences and Medicine, King’s College London, London, UK; 9 Centre for Gene Therapy and Regenerative Medicine, School of Basic and Medical Biosciences, Faculty of Life Sciences and Medicine, King’s College London, London, UK; 10 Department of Laboratory Medicine and Pathobiology, University of Toronto, Toronto, ON Canada; 11 Biomedical Research Centre, Guy’s and St. Thomas’ NHS Foundation Trust, London, UK; 12 Genomics Facility, Institute of Cancer Research, London, UK; 13 St John’s Institute of Dermatology, Guy’s, King’s, and St. Thomas’ Hospitals NHS Foundation Trust, London, UK; 14 Department of Plastic Surgery at Guy’s and St. Thomas’ NHS Foundation Trust, London, UK; 15 Department of Cancer Biology, Dana-Farber Cancer Institute, Boston, MA USA; 16 Department of Pathology and Laboratory Medicine, Mount Sinai Hospital, Toronto, ON Canada; 17 Department of Clinical Biochemistry, University Health Network, Toronto, ON Canada; 18 School of Cancer & Pharmaceutical Sciences, King’s College London, Guy’s Hospital, London, UK

**Keywords:** Tumour immunology, Melanoma, Lymphocyte differentiation, B cells

## Abstract

B cells are known to contribute to the anti-tumor immune response, especially in immunogenic tumors such as melanoma, yet humoral immunity has not been characterized in these cancers to detail. Here we show comprehensive phenotyping in samples of circulating and tumor-resident B cells as well as serum antibodies in melanoma patients. Memory B cells are enriched in tumors compared to blood in paired samples and feature distinct antibody repertoires, linked to specific isotypes. Tumor-associated B cells undergo clonal expansion, class switch recombination, somatic hypermutation and receptor revision. Compared with blood, tumor-associated B cells produce antibodies with proportionally higher levels of unproductive sequences and distinct complementarity determining region 3 properties. The observed features are signs of affinity maturation and polyreactivity and suggest an active and aberrant autoimmune-like reaction in the tumor microenvironment. Consistent with this, tumor-derived antibodies are polyreactive and characterized by autoantigen recognition. Serum antibodies show reactivity to antigens attributed to autoimmune diseases and cancer, and their levels are higher in patients with active disease compared to post-resection state. Our findings thus reveal B cell lineage dysregulation with distinct antibody repertoire and specificity, alongside clonally-expanded tumor-infiltrating B cells with autoimmune-like features, shaping the humoral immune response in melanoma.

## Abstract (web summary)

B cells are playing an active role in shaping the tumour immune microenvironment and the anti-tumour immune response in melanomas. Here authors show that intra-tumoral B cells are aberrantly activated and produce antibodies that are potentially autoreactive.

## Introduction

The importance and prognostic significance of T cells in cancer has been widely evaluated and well-established^1–3^. Yet it is only in the past decade that there has been a growing interest in understanding B cells, including their antigenic reactivities, isotype expression and relative contributions to tumor immune surveillance. Humoral immunity may play significant roles especially in immunogenic tumors such as malignant melanoma. B cells may accumulate at the tumor margin, infiltrate tumor lesions or form structures ranging from small cellular aggregates to more organized tertiary lymphoid structures (TLS). These features suggest that B cells are not simple bystanders, but may substantially influence the immune response, and consequently cancer progression and treatment outcomes^4–6^.

B cells are reported to have Janus-faced roles, as both suppressing or promoting tumor growth depending on the tumor microenvironment (TME), cellular phenotypes and antibody repertoires^7^. Previous studies by us and others have shown^4,5,8–11^ that, in immunogenic tumors including melanoma, certain aspects of B cell responses may be associated with differing disease outcomes^12^ and with response to immunotherapy^4–6^. Studies on serum samples show that dysregulated antibody isotype distribution featuring proportionally high IgG4 levels correlate with worse prognosis, while high IgG2 serum titers have been correlated with positive responses to checkpoint inhibitor treatment^13–15^. These suggest that the class switching process might play a role in clinical outcome. Bulk RNAseq transcriptomic analyses show an association between high immunoglobulin (Ig) expression/clonality and a more favorable prognosis^16,17^. However, high-throughput, bulk RNAseq based antibody repertoire analyses are often composed of short read sequences and might not provide information about isotype. Therefore, the study of intratumoral B cell repertoires to simultaneously evaluate variable region and matched isotype requires alternative methodologies such as long-read sequence analyses of Ig targeted polymerase chain reaction (PCR) from either whole tumor tissue or single cell sorted B cells. Several studies report the presence of tumor-specific but also self-reactive antibodies in the serum of cancer patients^18–22^ including melanoma^23–25^, where most appear to recognize intracellular antigens, such as melanocyte differentiation antigens (MDAs) and cancer-testis antigens^24–27^. However, although there is clear evidence for the involvement of B cells in cancer immunity, the current literature lacks comprehensive studies focused on the characterization of B cell phenotypes, intratumoral antibody repertoire and reactivity in the context of cutaneous melanoma.

In this study we employ mass cytometry (cytometry by time-of-flight, CyTOF), multicolor flow cytometry, immunofluorescence, immuno-mass spectrometry and transcriptomic analyses to characterize the B cell compartment in metastatic cutaneous melanoma. We conduct single cell sorting or tissue RNA extraction coupled with Ig-targeted PCR and long-read transcriptomic analyses to characterize tumor-associated B cell and antibody repertoires, including key B cell differentiation and maturation processes such as clonal expansion, somatic hypermutation (SHM), class switch recombination (CSR) and in situ receptor revision. We furthermore investigate antigen reactivities of antibodies derived from intratumoral IgD- memory B cells by immuno-mass spectrometry and glycan array analyses, alongside evaluation of antigen reactivity of circulating antibodies from patients by serum immuno-mass spectrometry. Overall, our study provides a comprehensive evaluation of the memory and class-switched B cells, including B cell phenotypes, antibody repertoire and specificity that shape the humoral immune response in metastatic cutaneous melanoma.

## Results

### Altered circulating memory and class-switched B cell populations in patients versus healthy volunteers

**Fig. 1:**
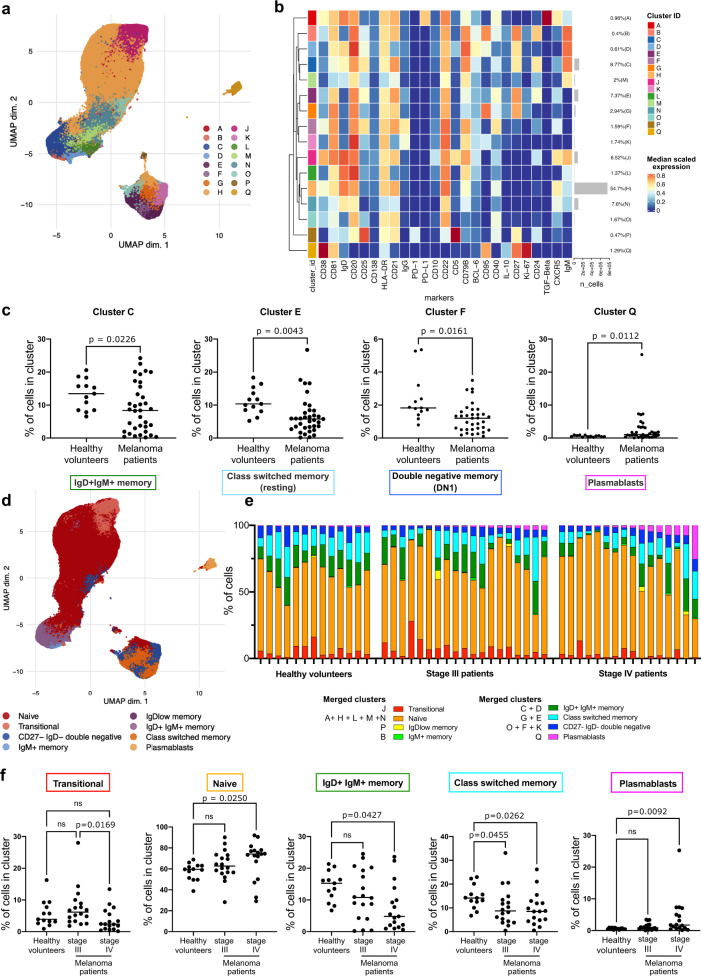
Altered proportion of memory B cell subsets and higher proportion of plasmablasts in melanoma patient compared to healthy volunteer blood. Evaluation of B cell subsets by CyTOF analyses (25 markers) (Biologically independent samples: Healthy volunteers, *n* = 13; Melanoma patients, *n* = 35). **a** UMAP plot for the B cell dataset showing the clusters generated using FlowSOM algorithm. **b** Heatmap representing the median scaled expression of the 25 markers used for B cell clustering. **c** Comparison of the relative abundance of differentially expressed B cell clusters in Healthy volunteers and Melanoma patients. **d** UMAP plot of the 16 clusters shown in **b** merged into 8 B cell populations. **e** Bar chart representing proportions of merged populations per sample. **f** Relative abundance of the 8 B cell populations in Healthy volunteers and Melanoma patients (divided per stage). Scatter plots representing mean and individual % of cells in cluster values. Statistical significance was calculated with two sided Welch’s test and nonparametric ANOVA; *P* < 0.05 are reported on the graphs. Source data are provided as a Source data file.

We investigated the B cell compartment in the circulation of patients with cutaneous melanoma (*n* = 35) and of healthy volunteers (*n* = 13) by CyTOF analyses with a panel of 25 B cell specific markers. We obtained 16 B cell metaclusters using FlowSOM analysis and we visualized them using a Uniform Manifold Approximation and Projection (UMAP) high-dimensionality reduction plot (Fig. 1a and Supplementary Fig. 1a) and heatmap to illustrate marker expression (Fig. 1b). We identified cell populations based on the median scaled expression of the markers used for B cell clustering, as summarized in Supplementary Table 1^28^. We evaluated differences in patients and healthy volunteer groups for each cluster using two statistical models: firstly by using a generalized linear mixed model Differential Abundance (DA) analysis, as previously described by Nowicka et al.^29^ (Supplementary Fig. 1b) and secondly by calculating the relative cell abundance per cluster in patients and healthy volunteers (Fig. 1c and Supplementary Fig. 1c). Within the 16 metaclusters, we found a significantly increased plasmablast compartment (cluster Q), and a decreased fraction of IgD-CD27- double negative memory cells (cluster F), representing double negative 1 (DN1) cells, in patients (Fig. 1c). These findings were confirmed by DA analyses (Supplementary Fig. 1b). We also reported that patients had a decreased proportion of cluster C and E, representing a fraction of IgD+IgM+ memory and resting class-switched memory B cells, respectively. To investigate whether the changes in specific compartments would be represented in the overall population we further merged the clusters into 8 B cell populations: transitional, naive, IgDlow memory, IgD+IgM+ memory, IgM+ only memory, class-switched memory, plasmablasts and IgD-CD27- memory (double negative) B cells (Fig. 1d–e). We also calculated the relative proportion of each of the 8 merged B cell populations in patients and healthy volunteers (Fig. 1e). Stratifying the patients by stage, we found significantly increased naive and plasmablast and decreased IgD+IgM+ memory and class-switched memory populations in patients compared to healthy volunteers (Fig. 1f). While for the class-switched memory populations the decrease compared to healthy volunteers was significant in both stage III and IV patients, the increase of naive and plasmablast and decrease of IgD+IgM+ memory populations was mainly driven by stage IV patients. We also found a significant increase of the transitional B cell compartment in stage III compared to stage IV patients.

These results suggest an overall decrease of the memory B cell compartment in the patient circulation, with reduced class-switched and IgD+IgM+ memory B cell populations, alongside increased antibody secreting plasmablast subsets in the peripheral blood of stage IV patients with melanoma.

### IgD- memory B cells accumulate at the tumor site

**Fig. 2:**
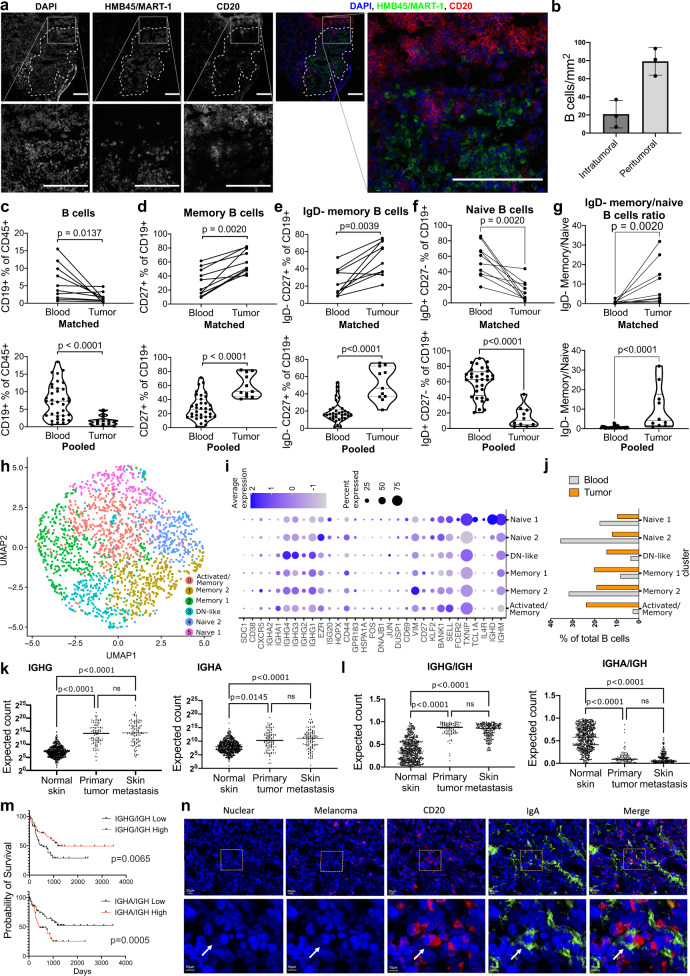
Memory B cells are enriched in the melanoma tumor microenvironment compared with peripheral blood. **a** Immunofluorescence images on melanoma tissue depicting cancer cells (HMB45/MART-1), and B cells (CD20): single colors (DAPI, HMB45/MART-1, CD20) and merged channels (DAPI, blue; HMB45/MART-1, green; CD20, red). Dotted line indicates tumor margins. Scale bar: 200 µm. **b** Intratumoral and peritumoral B cells per mm^2^ (biologically independent samples, *n* = 3). **c**–**g** Evaluation of B cells in patient blood compared with tumor samples by flow cytometry. Top panel: matched blood and tumor (*n* = 12); Bottom panel: pooled samples (blood, *n* = 45; tumor, *n* = 13). **c** B cells (CD19+ % of CD45+ lymphocytes). **d** Memory B cells (CD27+) % of B cells. **e** IgD- Memory B cells (IgD-CD27+) % of B cells. **f** IgG+ CD27- naive B cells. **g** IgD- memory/naive B cell ratio. **h**–**j** Single cell RNAseq analysis of matched blood and tumor. **h** UMAP visualization of B lymphocyte populations. **i** Expression of the cluster gene markers in each population. **j** Bar chart representing the relative abundance of each B cell cluster in blood and tumor samples. **k** IGHG (sum of *IGHG1*, *IGHG2*, *IGHG3*, *IGHG4*) and IGHA (sum of *IGHA1*, *IGHA2*) gene expression, and **l** IGHG/IGH and IGHA/IGH ratio (where IGH is the sum of IGHD, IGHM, IGHG, IGHA) in normal skin (*n* = 555), primary melanoma lesions (*n* = 102) and melanoma skin metastases (*n* = 116) (RSEM expected count (DESeq2 standardized) dataset, TCGA TARGET GTEx study, UCSC Xena). **m** Overall survival analysis for IGHG/IGH and IGHA/IGH ratio high (≥50 percentile) and low (<50 percentile). **n** Immunofluorescence images on melanoma tissue depicting cancer cells (HMB45/MART-1), B cells (CD20) and IgA: single colors, from left to right: DAPI, blue; HMB45/MART-1, pink; CD20, red; IgA, green; merged image (right). Statistical significance was calculated with two-sided Wilcoxon test (top panel) and Mann–Whitney test (bottom panel) (**c**–**g**), nonparametric ANOVA (**k**, **l**), Log rank (Mantel–Cox) (**m**); Error bars SEM of biologically independent samples, *n* = 3. (Biologically independent samples: Healthy volunteers, *n* = 13; Melanoma patients, *n* = 35; *P* < 0.05 are reported on the graphs. Source data are provided as a Source data file.

To study the presence and organization of B cells at the tumor site, we performed immunofluorescence staining on cutaneous melanoma metastases. B cells were identified by the expression of the pan-B cell marker CD20 (*n* = 7) (Supplementary Fig. 2a). To investigate the organization of B cells in the tumor area, cancer cells were stained using the known melanoma antigens HMB45 and MART-1 and the numbers of intratumoral and peritumoral CD20 + B cells were calculated (*n* = 3). B cells were mainly localized at the tumor edge but also formed small clusters inside tumors and in peritumoral areas (Fig. 2a, b). Accordingly, flow cytometric evaluations of tumor specimens (*n* = 17) showed that 64.7% had detectable B cells (Supplementary Fig. 2b, c). We analyzed matched blood and tumor (subcutaneous metastasis) samples from 10 melanoma patients (Fig. 2c–g, top panel) and pooled unmatched samples for a total of 34 patient blood and 11 tumor samples (Fig. 2c–g, bottom panel). These revealed a lower relative frequency of CD19 + B cells among CD45+ tumor-infiltrating cells compared to blood (Fig. 2c), consistent with reported low abundance of B cells in skin^8,30^. In matched and pooled cohorts, we found higher relative frequency of memory B cells (Fig. 2d) and IgD- memory B cells (Fig. 2e) in tumors compared to equivalent populations in patient blood. Furthermore, while the frequency of naive cells was decreased (Fig. 2f), the IgD- memory/naive B cell ratio was significantly increased in tumor compared to blood in comparative analyses of both matched and pooled samples (Fig. 2g).

Analyses from a publicly available single cell RNAseq dataset^31^ of a matched blood and tumor sample identified 6 B cell clusters (Fig. 2h) corresponding to naive, memory, activated/memory and double negative (DN)-like populations (Fig. 2i). Based on the relative abundance of blood and tumor B cells in each cluster, these analyses confirmed a decrease of naive clusters and an increase of the memory B cell compartments in tumor compared to blood (Fig. 2j), supporting our flow cytometry findings (Fig. 2d–g).

To gain an understanding of class-switched antibody expression in tumors, we investigated the expression of IGH genes in normal skin (*n* = 555), primary tumors (*n* = 102) and cutaneous melanoma skin metastases (*n* = 170) (TCGA TARGET GTEx (UCSC Xena)). We found increased total IgG and total IgA expression in primary tumors and skin metastases compared to normal skin, and comparable expression between primary tumor and skin metastasis (Fig. 2k). The IgG/Ig ratio was proportionally higher in both primary melanomas and skin metastases compared to normal skin, while the IgA/Ig ratio was lower (Fig. 2l), suggesting an overall humoral response activation. When we compared survival in relation to high (≥50th percentile) or low (<50th percentile) IgG/Ig or IgA/Ig ratios in skin metastatic lesions (*n* = 170) we found an association of high intratumoral IgG/Ig with increased survival probability, while higher intratumoral IgA/Ig associated with decreased overall survival probability (Fig. 2m). These results are in agreement with reports on the whole TCGA SKCM cohort^16^. We confirmed IgA antibody expression in melanoma lesions using immunofluorescence (Fig. 2n).

Our data confirmed that tumor-associated B cells mainly localized at the tumor margin and were observed infiltrating tumors in small clusters, and significant localization of IgD- (class-switched and IgM+) memory B cells and class-switched (IgG and IgA) antibody expression at the tumor site, which, depending on the isotype was positively or negatively associated with overall survival.

### Distinct variable region repertoires and antibody isotypes of tumor-associated memory B cells

**Fig. 3:**
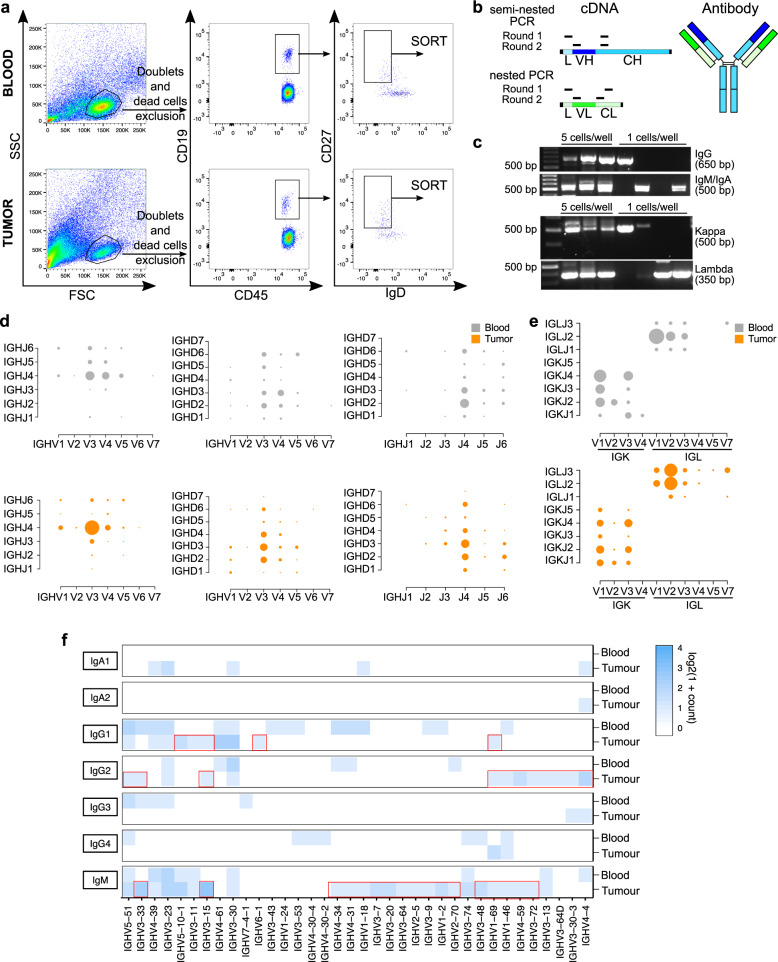
Antibody repertoires of IgD- memory B cells from melanoma patient blood and tumors. **a** Multicolor flow cytometry gating strategy for sorted IgD-CD27 + B cells from melanoma patients’ blood (top panel) and tumor (bottom panel). **b** Schematic showing the portions of the Ig sequences isolated: annealing position of the primers used for the semi-nested and nested PCR on the Ig heavy and light chain cDNA (Left panel) and corresponding sequences on a representative antibody structure (Right panel). **c** Agarose gel electrophoresis of the second round of PCR products for antibody heavy and light chains (Top panel: IgG or IgM/IgA; Bottom panel: kappa or lambda). **d**, **e** Bubble plots representing **d** the distribution of heavy chain IGHV-IGHJ, IGHV-IGHD and IGHD-IGHJ and **e** light chain IGKV-IGKJ and IGLV-IGLJ gene combinations, in melanoma patient’s blood and tumor samples. **f** Heatmap representing IGHV gene distribution grouped by isotype, in patient blood and tumor; tumor-specific IGHV-isotype combinations are highlighted in red. Source data are provided as a Source data file.

We sought to investigate the antibody repertoires of IgD- memory B cells in blood (*n* = 11) and tumors (*n* = 5) of melanoma patients by long-read sequence analysis, to obtain matched heavy and light chain sequences. We isolated IgD- memory B cells from patient blood and tumors either singly or in groups of 5–10 cells by flow cytometric sorting. We extracted the variable region and the beginning of the constant region sequences of the Ig heavy and light chains by PCR (Fig. 3a–c), to give information on both variable region and isotype in the same clone. We obtained 89 heavy and 92 light chain sequences from tumors and 57 heavy and 38 light chain sequences from patient blood. Of these, 26 and 7 matched heavy and light chain pairs from tumors and blood, respectively, were retrieved. Light chain and isotype distribution per patient are summarized in Supplementary Fig. 3a.

We analyzed the use of V, D, J genes in combination (Fig. 3d, e) and the relative frequencies of individual heavy (*IGHV*, *IGHD* and *IGHJ*) and light (*IGKV*, *IGKJ*, *IGLV* and *IGLJ*) chain genes (Supplementary Fig. 3b–d). These revealed significant differences in both V, D, J genes combinations and usage between patient blood and tumor compartments, pointing to distinct antibody responses in each compartment. We also annotated each variable region with its corresponding antibody isotype and analyzed the relative frequency of IGHV genes in blood and tumor by isotype. For some isotypes, particularly IgG1, IgG2 and IgM, we detected different groups of associated IGHV genes in blood versus tumor (Fig. 3f). This points to differential antigen reactivity of specific classes in the antibody repertoire of these compartments.

**Fig. 4:**
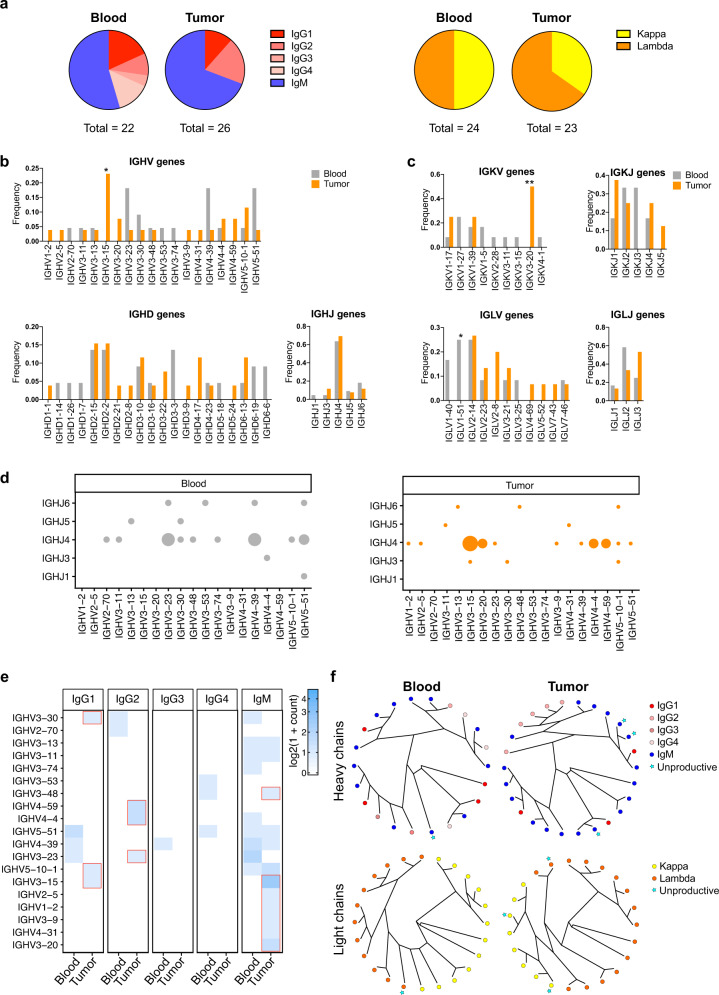
Antibody repertoire in patient’s matched blood and tumor. **a** Pie charts representing the isotype distribution of the heavy and light chain sequences isolated from blood and tumor of a patient with melanoma. **b** Frequency distribution of heavy chain IGHV, IGHD and IGHJ genes in blood and tumor. **c** Frequency distribution of kappa light chain IGKV and IGKJ genes and lambda light chain IGLV and IGLJ genes in blood and tumor. **d** Bubble plot representing the distribution of heavy chain IGHV-IGHJ gene pairing in melanoma patient blood and tumor. **e** Heatmap representing IGHV gene distribution grouped by isotype, in blood and tumor; tumor-specific IGHV-isotype combinations are highlighted in red. **f** Unrooted circular trees showing variable domain clustering according to sequence variability in blood and tumor heavy and light chain sequences annotated with information regarding the isotype or indicated if unproductive. Error bars represent bootstrapped 95% confidence intervals; statistical significance was calculated with a two-sample proportion *z*-test (**b**–**d**); *P* < 0.05 are reported on the graphs. * = *P* < 0.05, ** = *P* < 0.01. Source data are provided as a Source data file.

These differences in antibody repertoire between blood and tumor were further confirmed by our analysis on sequences from matched blood (21 heavy and 23 light chains) and tumor (25 heavy and 22 light chains, with 7 and 7 matched heavy and light chain pairs from tumors and blood, respectively) specimens of a patient with melanoma (Fig. 4a). We confirmed different V, D, J gene usage (Fig. 4b, c) and combinations (Fig. 4d) between these matched samples, supporting findings from the whole cohort. Consistent with analyses of the whole cohort, the relative frequency of IGHV genes by grouping per isotype (Fig. 4e) showed IgG1 and IgG2 to be associated with different IGHV genes in blood and tumor, and IgM to be associated with some IGHV genes in both blood and tumor, and others in tumor only. Furthermore, variable domain clustering represented by unrooted phylogenetic trees annotated with isotype, showed different clusters, with IgG2 clones associated with a particular VH cluster and IgG1 clones randomly associated with clusters in tumors, and more unproductive sequences in both heavy and light chains in tumor (11.5% and 6.9%) compared to blood (4.5% and 4.2%) (Fig. 4f).

Taken together, our results suggest the presence of a tumor-associated memory B cell compartment with distinct antibody repertoire and isotype distribution to that in the same patient’s circulation.

### Clonal expansion, SHM, CSR, receptor revision in tumor-infiltrating memory compartments

**Fig. 5:**
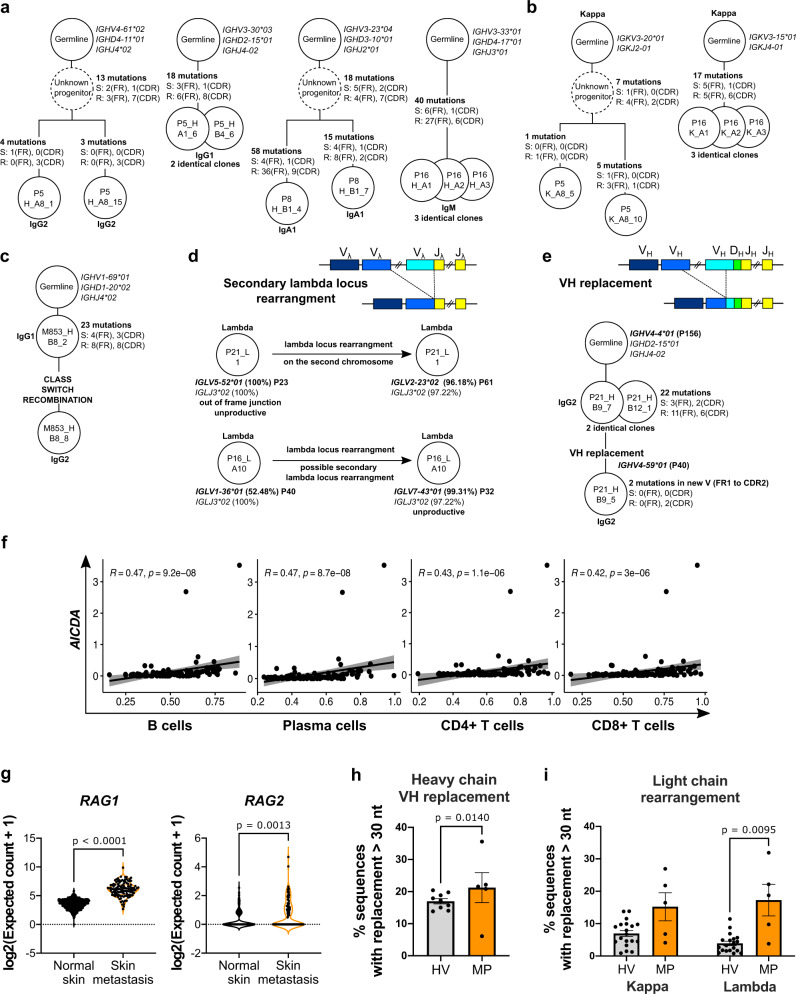
Tumor-associated B cells show evidence of in situ SHM, CSR and receptor revision. **a**, **b** Genealogical trees displaying examples of in situ SHM and clonal expansion in heavy (**a**) and light (**b**) chain of tissue resident B cell clones. **c** Genealogical tree displaying an example of in situ CSR. **d** Schematic representing secondary lambda locus rearrangement and examples of in situ lambda locus rearrangement. **e** Schematic representing VH replacement and genealogical tree representing an example of in situ VH replacement. **d**, **e** Germline genes are annotated with the relative position (P) in the locus. **a**–**e** Trees are annotated with mutations and isotype; mutations are annotated according to the following: Silent (S), Replacement (R), in Framework (FR), in CDR (CDR). **f**–**i** Long-read sequence analyses from bulk RNA extracted from tumor tissue. **f** Spearman correlation of *AICDA* gene expression with B cell, plasma cell, CD4 + T cell and CD8 + T cell signatures in melanoma skin metastases (TCGA dataset, *n* = 116) dataset. **g** *RAG1*, *RAG2* gene expression in normal skin (*n* = 555) and melanoma skin metastases (*n* = 116) (RSEM expected count (DESeq2 standardized) dataset, TCGA TARGET GTEx study, UCSC Xena). **h** VH replacement frequency in healthy volunteer blood (HV, *n* = 9 biologically independent samples) and melanoma patient tumor (MP, *n* = 5) derived from long-read sequence analyses. **i** Light chain rearrangement frequency in healthy volunteer blood (HV, *n* = 19 biologically independent samples) and melanoma patient tumors (MP, *n* = 5 biologically independent samples). The number of sequences in the high throughput repertoires analyzed in **h** and **i** are reported in Supplementary Tables 2 and  3. **g** Truncated violin plots: median (dashed black line), quartile (thin line). **h**, **i** Bar charts, error bars represent SEM. Statistical significance was calculated with the Kolmogorov–Smirnov test; *P* < 0.05 are reported on the graphs. Source data are provided as a Source data file.

To determine whether tumor-associated memory B cells actively respond to immune signals in situ, we investigated the presence of clonal expansion, SHM and CSR. Sequences derived from tumor-associated IgD- memory B cells (described above, Fig. 3) showed evidence of SHM, clonal expansion (Fig. 5a, b), and CSR from IgG1 to IgG2 (Fig. 5c). We found evidence of two lambda locus rearrangement events, a phenomenon not normally found in the periphery^32^, either on the homologous chromosome or on the same chromosome (Fig. 5d), in two single cell-sorted B cells expressing two lambda light chains, one productive and one non-productive, sharing the same JL family but different VL genes.

Furthermore, we found one example of IGHV (VH) replacement^33,34^, involving an upstream VH gene invading a cryptic heptamer in the VH gene of a VDJ rearrangement and resulting in a VDJ with a hybrid VH composed by the 5’ portion of the upstream VH and the 3’ portion of the old VH but unaltered DJ and therefore unaltered CDR3 (Fig. 5e). Pairwise alignment (Supplementary Fig. 4) showed that P21_B12_1 and P21_B9_5 sequences were similar to their own VH germline (92.7 and 95% similarity, respectively) and different from each other (82.5% similarity) until the end of the CDR2; after the CDR2 the sequences were identical to each other and showed more similarity to P21_B12_1 germline compared to P21_B9_5 germline (88.6% versus 87% similarity). This suggests that the recombination could have happened via the cryptic heptamer located at the end of the CDR2, and that IGHV4-59*01 gene has invaded the existing VDJ rearrangement (IGHV4-4*01, IGHD2-15*01, *IGHJ4-02*). Occurrence of lambda locus rearrangement and VH replacement in mature peripheral B cells are part of the receptor revision process which has been reported to be associated with autoimmunity^32,33,35^.

Based on evidence of significant expansion and differentiation of the humoral response in the TME, we investigated the expression of *AICDA* (*AID*, encoding the enzyme activation-induced cytidine deaminase, crucial for SHM and CSR), and of recombination-activating genes *RAG1* and *RAG2* (involved in V(D)J recombination) in normal skin (*n* = 555) and cutaneous melanoma skin metastases (*n* = 116) (TCGA TARGET GTEx (UCSC Xena)) (Fig. 5f–h). We found expression of *AICDA* in both normal skin and cutaneous melanoma (Supplementary Fig. 5) and a positive correlation of *AICDA* with B cell, plasma cells as well as CD4+ and CD8 + T cells signatures (Fig. 5f). We also reported a significant increase of the expression of *RAG1* and *RAG2*, genes known to be involved in B cell and T cell receptor rearrangements, in melanoma compared to normal skin (Fig. 5g), consistent with our findings of secondary lambda rearrangement and VH replacement^36^. To further explore evidence of receptor revision, we generated a long-read, high-throughput bulk RNA sequencing dataset from melanoma lesion samples (*n* = 5). In these, we investigated VH replacement and light chain rearrangements, in comparison with a dataset from healthy volunteers (heavy chain, *n* = 9^37^; light chain, *n* = 19^38^), according to Mallaby et al. method^39^. We confirmed higher prevalence of VH replacement (on average 21.26% of MP heavy chain sequences (*n* = 5, SD 10.44) vs 16.99% of HV sequences (*n* = 9, SD 2.44)) (Fig. 5h) and lambda locus rearrangement (on average 17.27% of MP lambda sequences (*n* = 5, SD 10.92) vs 3.91% of HV lambda sequences (*n* = 19, SD 2.87)) in intratumoral sequences (Fig. 5i).

Collectively, evidence of in situ SHM, clonal expansion and CSR, together with receptor revision, a process also reported in association with autoimmunity^36^, suggest an active IgD- memory B cell response with possible autoimmune and inflammatory features.

### Class-switched, affinity-matured and clonally-expanded antibodies associated with specific isotypes and polyreactive features at the tumor site

We investigated the B cell repertoire from long-read, high-throughput bulk RNA sequencing of B cells from melanoma tissues (*n* = 5). We obtained 27,506 unique heavy chain sequences and found a total of 2,886 clones composed by more than 1 unique sequence (Supplementary Table 2).

**Fig. 6:**
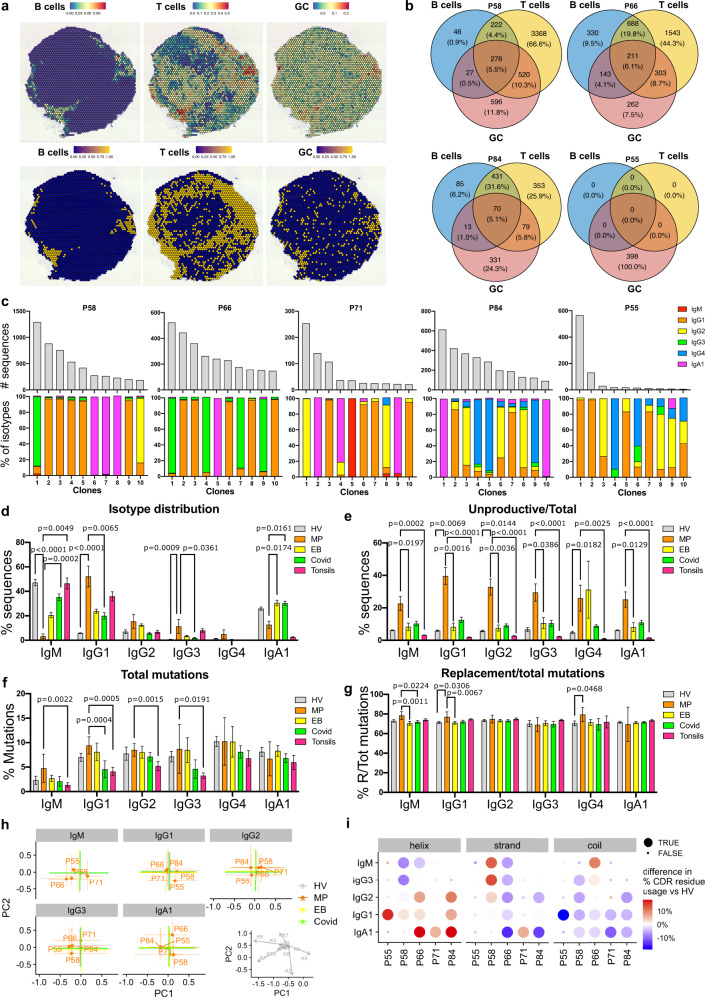
Spatial transcriptomic coupled with high throughput intratumoral antibody repertoire analyses suggest an active but aberrant B cell response. **a** Representative image of spatial transcriptomic deconvolution of B cells, T cells and B cell germinal center (GC) signatures. Top panels, signature score per spot; bottom panels, binarized values using 50% (B cells and T cells) and 85% (GC) threshold. **b** Venn diagrams representing the number of spots with B cells, T cells or GC signatures and their combinations. **c** Bar chart representing the absolute number of sequences (top panels) and the proportion of isotypes in the top 10 clones (bottom panels) showing CSR, per tumor. **d**–**g** Bar charts representing: **d** isotype distribution; **e** % of unproductive sequences; and **f** % of total mutations, and **g** % of replacement/total mutations in the V region of unique sequences from melanoma patients’ tumor (MP, *n* = 5) compared to healthy volunteers’ blood (HV, *n* = 9), ebola patient (EB, *n* = 12), SARS-CoV-2 patient (COVID-19, *n* = 16) and healthy tonsils (Tonsils, *n* = 8) repertoires. Statistical significance was calculated with non-parametric ANOVA compared to MP. Error bars SEM of biologically independent samples. **h** Principal component analysis (PCA) of heavy chain CDR3 characteristics in terms of Kidera factors. Dots depict median PC1/2 and colored lines depict inter-quartile range. **i** Comparison of the proportion of α-helical, β-strand and coil amino acid structures in the CDR3 sequences of the MP compared to HV repertoire data. *P* < 0.05 are reported on the graphs. Source data are provided as a Source data file.

To gain a better understanding on the involvement of tumor-infiltrating B cells in immune reactions at the tumor site, we investigated B and T cell associations by spatial transcriptomic evaluations (Visium, 10× Genomics) and with matched high throughput long read antibody repertoire analyses (*n* = 4). We aimed to investigate the presence of lymphoid aggregates and their maturity levels based on expression of germinal center genes and correlate these to the corresponding intratumoral antibody repertoire. We deconvoluted B cell and T cell populations per spot, alongside germinal center (GC) B cell signatures (Fig. 6a) and quantified the number of spots with coexisting B cells, T cells and GC signatures (Venn diagrams, Fig. 6b). Tumors with no (P55) or few (P84) lymphoid aggregates expressing germinal center signatures appeared to have fewer and smaller size B cell clones (Supplementary Fig. 6a) compared to the ones with a higher lymphoid structure-like signature (P58, P66). These point to spatial and likely functional links between B and T cells, with germinal center formation and antibody clonal expansion.

CSR within clones was detectable in five tumor samples analyzed and mainly to IgG1, IgG2 and IgA1 (Fig. 6c). We next analyzed melanoma lesion derived repertoires (MP) in comparison with repertoires from the blood of healthy volunteers (HV), recovered Ebola (EB) and SARS-CoV-2 (COVID) patients^37^, as well as a repertoire from tonsil samples (Tonsils)^40^, to provide a physiological setting which features activated B cells (Supplementary Table 3). In these analyses, we found differences in isotype distribution, with a decrease of IgM in melanoma patients compared to HV, COVID and Tonsils, a decrease of IgA1 compared to EB and COVID-19 and an increase of IgG1 and IgG3 compared to HV and COVID (Fig. 6d). We detected sequences which incorporated nonsense and frameshift mutations and became unproductive at a higher rate in melanoma that other datasets, such as tonsils (Fig. 6e). We found increased frequency of total mutations in melanoma compared to tonsils in IgM, IgG1 IgG2 and IgG3 (Fig. 6f). The Replacement/Total mutation ratio in melanoma was higher in IgM (against EB and COVID), IgG1 (compared to HV and EB) and IgG4 (compared with HV) (Fig. 6g).

To gain an insight into the antigen binding regions of the intratumoral expanded clones we compared the properties of their CDR3 amino acids (Kidera factors) with the above datasets (Fig. 6h, i). While HV, EB and COVID datasets showed similar CDR3 properties regardless of the isotype, the biochemical properties of the CDR3 regions of intratumoral clones differ from the other datasets (Fig. 6h). We therefore computed the proportion of amino acids within the CDR3 sequence which prefer α-helical, β-strand and coil structures and detected significant differences in structure propensities between MP and HV (Fig. 6i). We observed a significant increase in β-strand preference in IgA1 isotypes in one tumor, as well as for IgG2, IgG3 and IgM isotypes in a second tumor. We furthermore found a significant preference for α-helical structures for IgG1 (4 out of 5 tumors), for IgG2 (2 out of 5 tumors) and for IgA1 (3 out of 5 tumors) (Fig. 6i). Interestingly, β-strand-forming CDR3 is postulated as a hallmark of polyreactive antibodies^41^ and α-helical structures have been reported to be a key component in antibodies following affinity maturation^42^, together suggesting the presence of affinity-matured and promiscuous antibodies across different tumor specimens. We also report different *IGHV* gene distribution per different isotypes and in HV and MP datasets (Supplementary Fig. 6b, c). Of interest is V genes associated with autoimmunity appear to be preferred in certain sequence subsets in some tumors compared to both healthy volunteers’ blood and tonsil samples (e.g., *IGHV6-1*^43^: 85.71% of IgA1, patient P71; *IGHV1-69*^44^: 63.6% of IgM, patient P58, 65.9% and 31.5% of IgG1 in patient P55 and P58, respectively; IGHV4-34^44^: 42.1% of IgG2, patient P84; IGHV4-39^44^: 32.6% of IgM, patient P71). These support the hypothesis of polyreactive antibodies at the tumor site.

Together we report spatial and functional links between B and T cells, with germinal center formation and antibody clonal expansion, evidence of in situ clonal expansion SHM, with increased rate of replacement mutations in IgM, IgG1, IgG4, and CSR mainly to IgG1, IgG2 and IgA1. Alongside the higher rate in unproductive sequences, compared with the B cell active tonsil repertoire, and propensity for CDRH3 for structural properties characteristic of affinity-matured and polyreactive antibodies associated with specific antibody isotypes, support a highly active, class-switched, yet aberrant B cell compartment at the tumor site.

### Antigen discovery with patient-derived recombinant antibodies and circulating serum IgGs

**Fig. 7:**
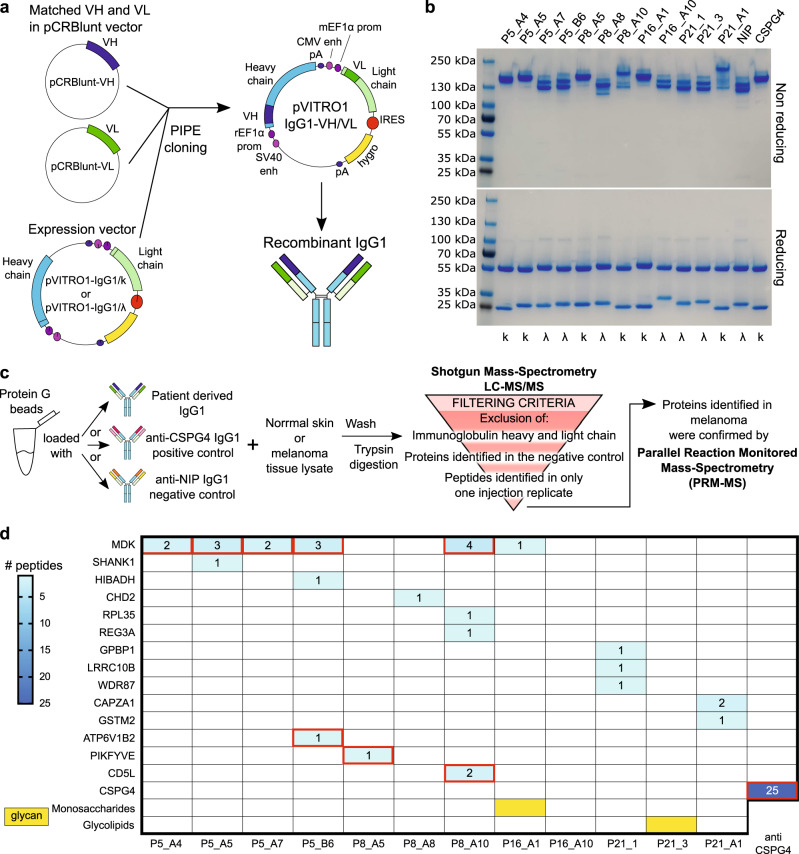
Patient-derived antibody production and antigen discovery. **a** Schematic explaining patient-derived antibody heavy and light chain cloning and production of recombinant human IgG1. **b** Coomassie SDS-PAGE of monoclonal antibodies bearing patient-derived variable region sequences in reducing and non-reducing conditions, annotated with kappa/lambda information. **c** Schematic of immuno-mass spectrometry experimental procedure and analysis pipeline for antigen discovery. Left panel: pull down of normal skin protein lysate or human melanoma protein lysate using patient-derived IgG1, anti-CSPG4 IgG1 (positive control) and anti-NIP IgG1 (negative control), followed by trypsin digestion and Shotgun Mass Spectrometry LC-MS/MS; Right panel: Shotgun Mass-Spectrometry LC-MS/MS output filtering criteria to select potential antigen peptides of interest to be confirmed by Parallel Reaction Monitored Mass Spectrometry (PRM-MS). **d** Summary of the immuno-mass spectrometry (blue boxes show the number of peptides pulled down by the antibodies from melanoma tissue protein lysate; red outlines represent an antibody reaction with a specific protein from normal skin protein lysate as well) and glycan array (yellow boxes show antibody reaction with specific glycans) screening results for antigen discovery with patient-derived antibodies. Source data are provided as a Source data file.

To investigate the antigenic reactivity of tumor-resident memory B cells, we generated twelve recombinant IgG1 antibodies from matched heavy (VH) and light chain (VL) variable regions, isolated by PCR from single cell sorted tumor-resident IgD- memory B cells. Matched sequences were PIPE-cloned into an expression vector carrying a human IgG1 backbone (pVITRO1-IgG1/k or pVITRO1-IgG1/λ) (Fig. 7a). IgG1 antibodies were expressed alongside the control (anti-melanoma recognizing the tumor antigen Chondroitin sulfate proteoglycan 4) anti-CSPG4 IgG1/k and anti-NIP (5-iodo-4-hydroxyl-3-nitrophenacetyl hapten-specific) IgG1/λ (Fig. 7b and Table 1: V region characteristics and original isotypes).

**Table 1:** Characteristics of the patient-derived antibodies we expressed as recombinant IgG1

Clone	Original isotype	Light chain	IGH			VH mutations		IGK/IGL		VL mutations	
			V	D	J	S	R	V	J	S	R
P5_A4	IgG2	k	V5-51	D1-26	J6	3	16	KV1-9	KJ3	3	11
P5_A5	IgM	k	V1-46	D1-26	J4	2	9	KV2-28	KJ4	3	7
P5_A7	IgM	λ	V3-74	D4-23	J4	2	7	LV1-51	LJ2	2	6
P5_B6	IgG1	λ	V5-10	D5-18	J2	4	16	LV2-11	LJ3	2	11
P8_A5	IgG2	k	V3-13	D6-19	J6	1	11	KV2-28	KJ1	3	11
P8_A8	IgM	λ	V3-30	D4-11	J6	5	10	LV1-51	LJ3	0	1
P8_A10	IgA2	k	V4-4	D2-8	J4	6	31	KV1-16	KJ4	9	2
P16_A1	IgM	k	V3-33	D4-17	J3	7	33	KV3-15	KJ4	6	11
P16_A10	IgM	λ	V1-69	D2-21	J6	2	5	LV7-43	LJ3	2	0
P21_1	IgM	λ	V3-9	D6-13	J4	2	8	LV2-23	LJ3	2	10
P21_3	IgM	λ	V3-20	D6-13	J4	4	10	LV2-14	LJ2	3	9
P21_A1	IgM	k	V3-48	D2-2	J6	1	2	KV3-20	KJ1	1	6

We investigated antigen reactivity of patient-derived antibodies for human proteins isolated from skin and melanoma tissue lysates by immuno-mass spectrometry (Fig. 7c for immuno-mass spectrometry pipeline) and for carbohydrates by glycan array. The positive control anti-CSPG4 IgG1 pulled down CSPG4 protein with 25 peptides in both normal skin and melanoma tissue (Fig. 7d). Of the 12 antibodies, 6 pulled down possible autoantigens from both skin and melanoma tissue, while 5 pulled down multiple proteins (Fig. 7d). Furthermore, comparing the binding to normal skin and melanoma, some antibodies were found to preferentially pull down proteins in skin rather than in melanoma, evidenced by the higher number of peptides detected by each antibody from skin-resident proteins (Supplementary Fig. 7). When we tested the melanoma lesion derived IgG1 antibodies for potential reactivity to several glycan families, two antibodies showed reactivity to carbohydrates, namely monosaccharides and glycolipids (Fig. 7d and Supplementary Figs. 8 and 9). These results suggest that among our patient-derived antibodies some recognize proteins expressed by melanoma cells and others likely react with glycans or glycoproteins expressed by melanoma cells.

**Fig. 8:**
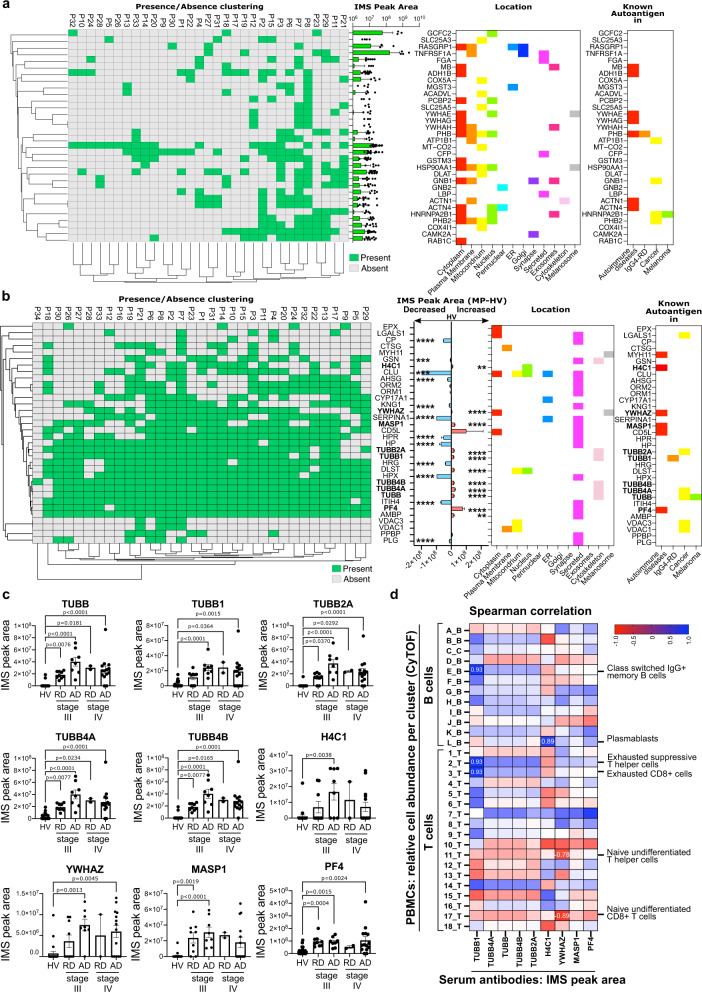
Immuno-mass spectrometry of serum immunoglobulins reveal altered autoantibody levels and reactivities in melanoma patients compared with healthy volunteers. **a**–**c** Biologically independent samples: melanoma patient (*n* = 33) and healthy volunteer (*n* = 23). **d** Biologically independent samples: melanoma patient (*n* = 7). **a**, **b** Serum immuno-mass spectrometry results representing proteins pulled down by antibodies from each serum sample. From left to right: clustered presence/absence heatmap flanked by a bar chart with **a** corresponding immuno-mass spectrometry (IMS) Peak Area depicting antigens pulled down by melanoma patient sera autoantibodies or **b** increase or decrease of IMS peak area in MP (*n* = 33) compared to HV (*n* = 23); antigen cellular or extracellular location; for known autoantigens: information on the disease setting. Proteins pulled down by **a** melanoma patients’ serum antibodies only, or by **b** both melanoma patients’ and healthy volunteers’ serum antibodies. In bold are the proteins of interest. **c** Analyses of IMS peak areas for proteins of interest (i.e., increased compared to healthy volunteers) representing the presence of autoantibodies in the serum samples (HV, healthy volunteers; patients with melanoma with stage III and stage IV disease (RD, resected disease; AD, active disease)). **d** Spearman correlation matrix of PBMC populations (relative cell abundance in B cell and T cell clusters analyzed by CyTOF) and serum autoantibodies (IMS Peak Area of the proteins in **c** of matched blood samples. Significant correlations (*P* < 0.05 and −0.5 <*r* < 0.5) are indicated by the presence of the *r* value on the heatmap box and by the description of the cell cluster. Statistical analyses were performed using non-parametric ANOVA (**c**) and Spearman correlation (**d**); error bars represent SEM of biologically independent samples. *P* < 0.05 are reported on the graphs. ** = *P* < 0.01, *** = *P* < 0.001, **** = *P* < 0.0001. Source data are provided as a Source data file.

To investigate and characterize a possible autoimmune response in melanoma, we tested the reactivity of melanoma patient (*n* = 33) and healthy volunteer (*n* = 23) serum IgG antibodies against a pool of tissue extracts via serum immuno-mass spectrometry^45–47^ (Supplementary Fig. 10). We identified 32 possible antigens recognized by autoantibodies present exclusively in melanoma patient (but not in HV) sera. Some of these are known autoantigens in autoimmune diseases^48–51^, IgG4 related disease (RD)^52^ and cancer^53–55^, including melanoma^56^ (Fig. 8a and Supplementary Fig. 11a). Furthermore, we identified autoantigens present in serum of both melanoma patients and healthy volunteers. Analysis of the peak area of each peptide’s signal showed some candidate autoantigens to be increased and others decreased in melanoma compared to HV sera: 11 significantly increased (TUBB, TUBB1, TUBB2A, TUBB4A, TUBB4B, H4C1, CYP17A1, YWHAZ, MASP1, DLST, PF4, AMBP) and 14 significantly decreased (CP, PLG, GSN, CLU, AHSG, ORM1, ORM2, KNG1, SERPINA1, HRG, HPX, ITIH4, HPR, HP) in patients. This may point to the presence of an altered baseline autoimmune response for certain antigens in patients (Fig. 8b and Supplementary Fig. 11b). We then stratified the patients per stage (stage III, stage IV) and disease status (resected or active disease). We found significantly higher autoantibody levels against nine antigens (TUBB, TUBB1, TUBB2A, TUBB4A, TUBB4B, YWHAZ, MASP1, H4C1 and PF4) in patients with active disease at the time of sampling compared to patients with resected disease and HV (Fig. 8c). TUBB, TUBB1, TUBB2A, TUBB4A and TUBB4B belong to the tubulin family, and gene expression analyses showed a higher expression of all these tubulins in melanoma compared to normal skin (Supplementary Fig. 12). Furthermore, YWHAZ, MASP1, H4C1 and PF4 have been reported in association with autoimmune diseases^48–51^.

To evaluate links between autoreactive antibodies with B and T cells, for the serum samples analyzed by immuno-mass spectrometry, we performed CyTOF analysis on the matched PBMCs from 7 patients with stage III, active disease, selected because autoantibody responses were significantly enhanced in active disease compared with resected disease in our cohort (Supplementary Fig. 13). We then conducted Spearman correlation of autoantibodies targeting tubulins as well as YWHAZ, MASP1, H4C1 and PF4 with B and T cell subsets identified by CyTOF (Fig. 8d). TUBB1 autoantibody reactivity showed a significant positive correlation with a B cell cluster of switched IgG+ memory B cells (cluster E_B), consistent with serum IgG class autoantibodies to TUBB1. TUBB1 autoreactivity also correlated with two T cell clusters: exhausted suppressive T helper cells (cluster 2_T), and suppressive-like exhausted CD8 T cells (cluster 3_T). These analyses also revealed a positive correlation of antibodies targeting H4C1 histone protein and IL-10 expressing plasmablasts in patient blood.

Together, these findings suggest perturbed autoantigen-reactive humoral responses in melanoma, especially in patients with active disease.

## Discussion

We conducted in-depth cell subset and antibody repertoire analysis of circulating and tumor-resident B cells in metastatic cutaneous melanoma, antigen discovery with melanoma patient serum samples and characterization of recombinant antibodies derived from tumor-resident IgD- memory and from total B cells.

We report an overall decrease of the memory B cell compartment and a corresponding increase of antibody-secreting plasmablasts in the peripheral blood of stage IV patients with melanoma as compared with healthy volunteers. This may denote, in melanoma patients, potential activation of antibody secreting B cells with a simultaneous decrease of memory B cells from the peripheral blood. In contrast, comparing the B cell compartment in patients’ blood and tumor by flow cytometry and single cell RNAseq analyses revealed higher relative frequencies of both memory B cells and IgD- memory B cells in tumors compared to blood. In line with recent findings in the B cell compartment of breast cancer patients^57^, here we show an increased IgD- memory/naive B cell ratio in tumors compared to melanoma patient circulation, suggesting a bias towards class-switched humoral responses in the TME. Consistent with published reports of tertiary lymphoid structure assembly in melanoma, immunofluorescence staining of cutaneous metastases revealed infiltrating B cells forming small clusters mostly localized at the tumor edge as well as within tumor islets^4,58^. We report the presence of lymphoid aggregates expressing GC B cell signatures, a positive correlation with the number and size of antibody clones, and links between B cells with T cells associated with germinal center formation and antibody clonal expansion.

We investigated intratumoral antibody repertoires using two datasets: a low throughput dataset from sorted IgD- memory B cells from melanoma patient blood and tumors and a high throughput dataset from tumor tissues. Antibody repertoires of IgD- memory B cells in patient blood and tumors generated by single cell sorting, coupled with Ig targeted PCR and sequencing, were obtained from long reads covering the whole variable region and the beginning of the constant region to simultaneously study variable region and matched isotype and to match antibody heavy and light chain for patient-derived antibody development. In agreement with our previous findings^8^, we found distinct V, D, J variable region gene usage and pairing in tumor compared to blood, an observation further confirmed in matched blood and tumor samples. Annotating each variable region with the corresponding isotype revealed that some groups of IGHV genes were preferentially associated with specific antibody isotypes, which in line with emerging evidence that antibody isotype can affect an antibody’s function and affinity for its target antigen^59^, suggest the selection of IgD- memory B cells with a specific antibody variable region and isotype repertoire at the tumor site, which appears distinct from that in the circulation. High throughput sequencing of tumor-associated antibodies derived from all B cell types, including plasma cells, confirmed unique characteristics of the tumor associated class-switched B cell compartment. In both datasets we found evidence of in situ clonal expansion, and CSR to IgG1, IgG2 and IgA1 and SHM. This is further supported by a positive correlation of AICDA gene expression and B cell, plasma cell, CD4+ and CD8 + T cell profiles in melanoma skin tumor metastases, which could also indicate B and T cell crosstalk giving rise to plasma cell differentiation. We also showed a higher rate in unproductive sequences, compared with a tonsil dataset representing active B cells involved in a physiological immune reaction. These point to a distinct tumor-resident active, class-switched, yet aberrant humoral response.

Several lines of evidence support expression of antibodies with polyreactive features in melanoma. CDRH3 physiochemical properties were favored in intratumoral compared to healthy volunteer blood B cell repertoires, especially for IgG2, IgM and IgA1. IgG1, IgG2 and IgA1 CDRH3 showed preference for α-helical structures, associated with affinity maturation^42^, while for some tumors, intratumoral IgM, IgG2, IgG3 and IgA1 CDRH3 sequences showed a significant increase in β-strand preference, which is postulated as a hallmark of polyreactive antibodies^41^. These support the presence of affinity-matured and promiscuous antibodies across different tumor specimens. A link between tumor-associated antibody responses and autoimmunity is further supported by the expansion of clones carrying the *IGHV1-69*, *IGHV4-34*, *IGHV4-39*, *IGHV6-1* genes, associated with autoimmunity^43,60,61^, in intratumoral antibodies compared to both healthy volunteers’ blood and tonsil samples.

In situ VH replacement and lambda locus rearrangement events in both IgD- memory B cell and high throughput melanoma tissue antibody repertoires, are thought to be part of the receptor revision processes occurring in mature B cells in the periphery and often associated with autoimmunity^32,33,35^. Consistent with evidence of receptor revision are our observations of elevated intratumoral expression of *RAG1* and *RAG2* genes, crucial for V(D)J recombination and usually not expressed in mature immune cells or in the periphery and which could be part of a deregulated immune response^62^. It is possible that receptor revision rearrangement, as well as affinity maturation, occur before migration of B cells to the tumor. However, increased VH replacement and lambda locus rearrangement events in tumors compared to blood, may create conditions for these events to occur at the tumor site. To our knowledge ours is the first reported evidence of receptor revision in melanoma associated B cells. This could have been overlooked by clonotype analysis procedures which impose identical VDJ combination as a criterion for defining antibody clones. These phenomena are linked to BCR revision and are known to be involved in autoimmunity^35^.

We generated recombinant antibodies derived from tumor-resident IgD- memory B cells and we investigated their antigen reactivity against human cutaneous and melanoma proteins, by immuno-mass spectrometry, a proteome-wide method to identify antibody targets, and carbohydrates by glycan array. Immuno-mass spectrometry data show that our patient-derived antibodies bind to some melanoma-associated proteins but also proteins present in normal skin or to multiple proteins. The glycan array shows that 17% of the antibodies bind to some glycans, mainly monosaccharides and glycolipids. Of the antibodies we cloned, only one was originally an IgG1, the rest had IgG2, IgM or IgA2 isotypes, therefore we cannot exclude the possibility that expressing these antibodies as IgG1 could have influenced their reactivity. Our conclusions are limited by the low number of antibodies produced, but are consistent with the fact that although antibody discovery technology is widely available, very few reports of tumor-specific antibodies from patients with melanoma have reported definitive results. Of the existing reports to-date, most antibodies derived from cancer patients appear to recognize intracellular antigens, such as MDAs and cancer-testis antigens^24–27^. Furthermore, our patient-derived antibodies were generated from clonally-expanded cells with possible autoimmune like features. It is therefore perhaps not surprising that immuno-mass spectrometry and glycan array showed some reactivity and polyreactivity with antigens found in both normal skin and melanoma.

Further supporting an autoreactive immune response, immuno-mass spectrometry screening of circulating serum IgGs revealed autoantibodies present exclusively in melanoma patients. Most of these antibodies recognized antigens associated with autoimmune diseases, IgG4 related disease and cancer. Differential levels of autoantibodies in patients compared with healthy subjects suggest an altered physiological baseline autoimmune response for certain antigens in melanoma. Of particular interest, anti-tubulin autoantibodies and antibodies to YWHAZ, MASP1, H4C1 and PF4 were significantly higher in patients with active disease at the time of sampling, compared to patients with resected disease in comparison with healthy volunteers. Their increase appears in most of the patients, regardless of autoimmune disease co-morbidity, suggesting that perturbed levels of autoantibodies in melanoma are likely related to the cancer condition and active disease rather than to pre-existing autoimmune disorders. Furthermore, our data from matched serum and PBMCs samples, from patients with active disease, show that TUBB1 autoantibodies positively correlate with class-switched IgG+ memory B cell and with exhausted T helper and CD8 + T cell subsets, and that autoantibodies to H4C1 positively correlated with plasmablasts. Autoantibodies targeting histone proteins such as H4C1 have been reported to be associated with systemic lupus erythematosus^63^. Autoreactivity to TUBB and TUBB2A has been reported in cancer^64^, and only TUBB has been reported in melanoma^65^. In concordance with these observations, the gene expression of these tubulins was significantly increased in melanoma compared to normal skin. Consistent with levels of lactate dehydrogenase release, a well-described biomarker of active disease in melanoma, we observed increased levels of tubulins which may also be associated with melanoma burden. Higher expression of these could explain the increase of anti-tubulin autoantibodies in our melanoma cohort with active disease. The possible link between anti-tubulin autoantibodies and active disease is of particular interest from a biomarker perspective and merits further investigation.

The analogy between cancer and autoimmune lesions has been widely discussed and systemic immune reactions to self-antigens in melanoma have long been reported^66–69^. The possibility of an autoimmune like response at the tumor site could explain the spontaneous occurrence of vitiligo in 1–3% of melanoma patients^70^ as well as the development of vitiligo in melanoma patients treated with immune checkpoint inhibitors and cell based immunotherapies^71^. The development of vitiligo in advanced melanoma may suggest abnormal immune destruction of melanocytes sharing common antigens with melanoma cells. The occurrence of vitiligo is largely correlated with a more favorable prognosis^66,70^ and insurgence of vitiligo in immune checkpoint inhibitor treated patients is associated with a substantial reduction of both disease progression and death risks^71^; this has been recently defined as beneficial autoimmunity^72^. However, these phenomena have not been sufficiently studied in relation to the B cell response in melanoma lesions.

In conclusion, focusing on the humoral B cell response in cutaneous metastatic melanoma we report evidence of a heightened IgD- class-switched memory B cell response at the tumor site compared to patient circulation and differential antibody repertoire and isotype distribution in blood and tumor, confirmed in matched blood and tumor analyses. We show spatial and functional links between B and T cells, with germinal center formation and antibody clonal expansion, SHM, CSR and receptor revision, as well as propensity for CDRH3 properties characteristic of affinity-matured and polyreactive antibodies, which support a highly active yet perturbed B cell compartment at the tumor site. Alongside, antigen discovery with cloned tumor-resident memory B cell derived antibodies and circulating serum IgGs show some recognition of autoantigens and evidence of an altered baseline autoimmune response for certain antigens in melanoma patients, with some of these autoantibody features associated with active disease. Our study provides a new perspective of the aberrant nature of B cell phenotypes, antibody repertoire and specificity, which shape the humoral immune response in metastatic cutaneous melanoma and highlights autoimmune signatures for clonally-expanded B cell repertoires.

## Methods

### Human samples collection

Human peripheral blood and tumor tissue samples were collected with informed written consent in accordance with the Declaration of Helsinki. The study was conducted at King’s College London, Guy’s and St Thomas’ NHS Foundation Trust (REC reference: 08/H0804/139 approved by London Bridge NRES committee; REC reference: 16/LO/0366 approved by London-Central NRES Committee). Blood was collected: from 35 patients with cutaneous melanoma (Supplementary Table 4), and from 13 healthy volunteers (Supplementary Table 5) (CyTOF analyses); from 29 patients with cutaneous melanoma (Supplementary Table 6) (Flow Cytometry analyses). Furthermore, 17 melanoma samples (17 cutaneous and subcutaneous metastases) were obtained (Supplementary Table 7). Blood for serum analyses was collected from 33 patients with cutaneous melanoma (Supplementary Table 8) (immuno-mass spectrometry analyses). All the patients were immunotherapy treatment naive. Patients were staged and classified according to the American Joint Committee on Cancer Melanoma Staging and Classification criteria^73,74^.

### Human cell isolation

Peripheral blood mononuclear cells (PBMCs) were isolated from 40 ml blood (or leukocyte cones) using Ficoll® Paque Plus density centrifugation (GE Healthcare). Melanoma and skin tissue were minced with a scalpel and then mechanically dissociated with gentleMACS dissociator in RPMI 1640 medium supplemented with 1 mM EDTA. The cell suspension and the remaining pieces of tissue were then incubated overnight at 37 °C, 5% CO_2_ in RPMI 1640 medium supplemented with 10% heat inactivated Fetal Bovine Serum (FBS) and Penicillin-Streptomycin (10,000 U/ml) to allow the remaining immune cells to crawl out of the tissue. The cell suspension was then harvested, passed through a 100 µm cell strainer, and processed for B cell phenotyping.

### Mass cytometry (CyTOF) analysis

PBMCs were washed in MaxPar Cell Staining Buffer (Fluidigm) at 800 g for 5 min and cells were incubated Fc-Blocking Solution (Human Trustain Fc blocking solution (Biolegend)) for 10 min at room temperature. A cell surface staining antibody cocktail of metal-tagged antibodies was added and incubated 30 min on ice, followed by 194 Cisplatin (Fluidigm) live/dead staining. The cells were washed as above, fixed using Fix I buffer (Fluidigm), and permeabilized using Perm S buffer (Fluidigm), followed by staining with an intracellular staining cocktail for 30 min at room temperature. Antibodies are listed in Supplementary Table 9. After washing, cells were incubated overnight at 4 °C with intercalation solution consisting of DNA intercalator 103-Ir (Fluidigm) and Fix + Perm Buffer (Fluidigm), washed once with MaxPar Cell staining Buffer (Fluidigm) and twice in MilliQ water. EQ Calibration beads were added as per the manufacturer’s directions prior to running. Data were acquired on a Helios mass cytometer (Fluidigm).

B cells (CD45 + CD19 + CD3-CD4-CD8-) population was gated using FlowJo software (Supplementary Fig. 1a) and.fcs files were uploaded in R. A selection of 25 B cell directed phenotypic markers (Fig. 1b) was used to perform in-depth B cell phenotyping. We used a modified R script based on the CATALYST, diffCYT, FlowSOM, edgeR and flowCORE packages which can be found using the bioconductor terminal https://www.bioconductor.org/packages/release/bioc/vignettes/CATALYST/inst/doc/differential.html. Unsupervised clustering of B cell populations was performed using FlowSOM package. High dimensionality reduction was performed for data visualization in a 2D plot using a UMAP algorithm and a heatmap was used to visual median marker scaled expression.

For T cells analysis, samples were gated in FlowJo (BD, version 10.8.1) where single cells, CD45+, CD20-, CD19-, CD16- and CD3+ were exported as single FCS files. Samples were then imported on the online platform Cytobank (https://www.cytobank.org), where an unsupervised analysis was performed for the dimensionality reduction step projecting the data onto uniform manifold approximation and projection (UMAP)^75^. New FCS files with UMAP1 and 2 coordinates were exported, and cells were clustered into groups of phenotypically similar cells using clustering algorithm PhenoGraph with a custom script implemented on R (available to download here https://github.com/JinmiaoChenLab/Rphenograph^76^). Phenotypical markers involved in T cells subsets’ characterization, activation and proliferation were used for the clustering: CD4, CD8, CD25, CD5, CD27, CD81, CD38, BCL6, CD28, Ki67, HLA-DR and CD279 (PD-1).

### Multicolor flow cytometry analysis and cell sorting

PBMC and tumor single cell suspensions were stained with LIVE/DEAD Fixable Aqua (Invitrogen) according to the manufacturer’s instructions and then incubated with Fc Blocking Reagent (Miltenyi Biotec) for 10 min at room temperature followed by staining with anti-CD45 PerCP (BioLegend), anti-CD19 FITC (BD), anti-CD27 BV421 (BD) and anti IgD APC H7 (BD) (BioLegend) for 30 min at 4 °C. The samples were then washed in Phosphate-Buffered Saline (PBS) 2% FBS (FACS Buffer), resuspended in 200 µl of FACS Buffer and analyzed by multicolor flow cytometry using BD FACSCanto II (BD Biosciences). Tumors without detectable B cells (less than 10 per sample) were excluded from analyses. For Ig sequence analysis, CD27+IgD- B cells (CD45 + CD19+) were sorted on 96-well plates at 1, 5, and 10 cells/well directly into 10 µl of lysis buffer (Single cell lysis kit, Ambion-Thermo Fisher Scientific), using BD FACSAria II Cell Sorter (BD Biosciences) with a 70 µm nozzle.

### Immunofluorescence staining of human melanoma tissue samples

OCT embedded frozen melanoma tissue was cut using Leica cryostat to produce 4 µm thick slices. Slices were fixed with 4% formaldehyde (w/v), methanol-free (Pierce) for 5–10 min and washed 2 times in PBS for 5 min. Samples were then incubated in Blocking Buffer (5% FBS, 0.1% Triton X-100 in PBS) for 1 h at room temperature, followed by incubation with primary antibodies: rabbit monoclonal anti-CD20 and mouse monoclonal anti-melanoma antibody mix (anti-HMB45 + anti-MART-1 M2-7C10 and M2-9E3) overnight at 4 °C. The slides were then rinsed in Washing Buffer (0.1% Triton X-100 in PBS) and incubated with secondary antibodies: pre-adsorbed Goat Anti-Rabbit IgG H&L (Alexa Fluor® 568) and pre-adsorbed Goat Anti-Mouse IgG H&L (Alexa Fluor® 488) for 1 h at room temperature. Samples were washed as above and mounted using ProLong Gold Antifade Mounting medium with DAPI (Thermo Fisher Scientific). Images were acquired using Nikon A1 Inverted Confocal microscope, we thank the Nikon Imaging Centre at Kings College London for help with light microscopy.

### Transcriptomic analysis in normal skin and melanoma

RSEM expected count (DESeq2 standardized) dataset from TCGA TARGET GTEx study (UCSC Xena) was used to compare gene expression in normal skin (*n* = 555), melanoma skin metastases (*n* = 116), lymph node metastases (*n* = 208), visceral metastases (*n* = 36). Data wrangling was performed with RStudio (Version 1.3.1093), plots and statistical analysis were generated with GraphPad Prism software (version 9, GraphPad).

### Single-cell RNAseq

Single-cell RNA-seq data from a matched blood and tumor of a treatment-naive patient was obtained from the publicly available dataset GSE123139^31^. Cluster containing B cells (*n* = 1867, markers: CD79A, CD79B, MS4A1) was identified and used for downstream analysis and annotation using Seurat package (4.0.6)^77^.

### Generation of IgD- memory B cell Ig sequences dataset

Samples with sorted and lysed IgD- memory B cells, obtained as described above, were used for reverse transcription with SuperScript VILO cDNA Synthesis kit (Thermo Fisher Scientific) according to the manufacturer’s recommendations. The variable region sequences of Ig heavy and light chains were amplified by semi-nested and nested PCR respectively, using Phusion Flash High-Fidelity PCR Master Mix (Thermo Scientific) as in^78,79^. PCR products were cloned into pCR-Blunt vector using ZeroBlunt PCR cloning kit (Invitrogen). Plasmid DNA was purified using QIAprep spin miniprep kit (Qiagen) and sequenced by Sanger sequencing (Source Bioscience) using the M13 forward primer.

### Generation of high-throughput melanoma Ig sequences dataset and Visium spatial transcriptomics

OCT frozen tumor sample (*n* = 5) were processed through 10X Genomics Visium platform for spatial transcriptomic analyses according to manufacturer’s instructions. For generation of matched high throughput long reads antibody repertoires, a 2 µl sample of cDNA from Visium experiments was processed through a Read1 step-out PCR priming on variable gene of the heavy and light chains at the 3’ and the Read1 sequence added at the 5’. The sample was cleaned with SPRIbeads at ×0.8 using a left-sided selection, eluted into 35 µl of TE buffer. The second PCR used step-out primer landing sites added in PCR to increase specificity and yield using the full 35 µl elute. Primers are listed in Table 2. Both PCRs used 100 µl reactions of NEB Q5 high fidelity enzyme, according to the manufacturer’s instructions with the GC enhancer, with the following reaction conditions: 30 s at 98 °C, 15×[10 s at 98 °C, 30 s at 65 °C, 60 s at 72 °C], 5 min at 72 °C. The sample was cleaned with SPIRbeads with a ×0.8 left sided selection and sent for sequencing at University of Liverpool CGR using PacBio Sequel 2 technology.

**Table 2:** Primers for tissue Ig long read PCR

Primer name	Stage	Sequence 5’–3’
10X Step-out	PCR1-All	CACTCTATCCGACAAGCAGTCTACACGACGCTCTTCCGATCT
Visium_VH1_F	PCR1-Heavy	GTGACTGGAGTTCAGACGTGTCCATGGACTGGACCTGGA
Visium _VH2_F	PCR1-Heavy	GTGACTGGAGTTCAGACGTGTCAGATGGACATACTTTGTTCCAC
Visium _VH3_F	PCR1-Heavy	GTGACTGGAGTTCAGACGTGTCCATGGAGTTTGGGCTGAGC
Visium _VH4_F	PCR1-Heavy	GTGACTGGAGTTCAGACGTGTCGATGAAACACCTGTGGTTCTT
Visium _VH5_F	PCR1-Heavy	GTGACTGGAGTTCAGACGTGTATGGGGTCAACCGCCATCCT
Visium _VH6_F	PCR1-Heavy	GTGACTGGAGTTCAGACGTGTGATGTCTGTCTCCTTCCTCAT
L_Vk1/2_F	PCR1-Kappa	GTGACTGGAGTTCAGACGTGTATGAGGGTCCCCGCTCAGCTGCTGG
L_Vk3_F	PCR1-Kappa	GTGACTGGAGTTCAGACGTGTCTCTTCCTCCTGCTACTCTGGCTCCCAG
L_Vk4_F	PCR1-Kappa	GTGACTGGAGTTCAGACGTGTATTTCTCTGTTGCTCTGGATCTCTG
L_Vλ1_F	PCR1-lambda	GTGACTGGAGTTCAGACGTGTGGTCCTGGGCCCAGTCTGTGCTG
L_Vλ2_F	PCR1-lambda	GTGACTGGAGTTCAGACGTGTGGTCCTGGGCCCAGTCTGCCCTG
L_Vλ3_F	PCR1-lambda	GTGACTGGAGTTCAGACGTGTTCTTATGAGCTGACACAGCCA
L_Vλ4/5_F	PCR1-lambda	GTGACTGGAGTTCAGACGTGTGGTCTCTCTCGCAGCCTGTGCTG
L_Vλ6_F	PCR1-lambda	GTGACTGGAGTTCAGACGTGTGTTCTTGGGCCAATTTTATGCTG
L_Vλ7_F	PCR1-lambda	GTGACTGGAGTTCAGACGTGTGGTCCAATTCCCAGGCTGTGGTG
L_Vλ8_F	PCR1-lambda	GTGACTGGAGTTCAGACGTGTGAGTGGATTCTCAGACTGTGGTG
Step-PID1-Sequel	PCR2-Forward	GGTAGCACATATCAGAGTGCGCACTCTATCCGACAAGCAGT
R2-PID1-Sequel	PCR2-Reverse	CCATCCACATATCAGAGTGCGGTGACTGGAGTTCAGACGTGT

### Antibody repertoire analysis

Full-length antibody sequences from the IgD- memory B cell dataset were collected and annotated using IMGT/HighV-QUEST^80,81^. Heavy chain subclasses were assigned by aligning the collected sequences with IGHC reference sequences obtained from IMGT using the BWA-MEM algorithm (version 0.7.17.1 on Galaxy [https://usegalaxy.org]) (https://arxiv.org/abs/1303.3997). Annotation of CDR3 Kidera features was performed using the BRepertoire webserver^82^. Sequence clustering was performed on BRepertoire using the entire V(D)J nucleotide sequence and visualized as circular trees. Data visualization was produced either using the BRepertoire webserver or in-house scripts under the R statistical programming environment (v3.6.2). The following statistical analyses were performed: first, for comparison of gene usage frequencies, a two-sample proportion *z*-test was used, following ref. ^83^. Briefly, to compare two proportions *p*_T_ (over *n*_T_ sequences) and *p*_B_ (over *n*_B_ sequences) we computed these quantities:

1 $$\hat{p}=\frac{{n}_{{{{{{\rm{T}}}}}}}{p}_{{{{{{\rm{T}}}}}}}+{n}_{{{{{{\rm{B}}}}}}}{p}_{{{{{{\rm{B}}}}}}}}{{n}_{{{{{{\rm{T}}}}}}}+{n}_{{{{{{\rm{B}}}}}}}}$$

2 $$Z=\frac{{p}_{{{{{{\rm{T}}}}}}}-{p}_{{{{{{\rm{B}}}}}}}}{\sqrt{\hat{p}(1-\hat{p})(\frac{1}{{n}_{{{{{{\rm{T}}}}}}}}+\frac{1}{{n}_{{{{{{\rm{B}}}}}}}})}}$$

The value of *Z* was compared against the standard normal distribution to determine statistical significance. Secondly, for comparison of ratios of antibody class/subclass across different data groupings, the observed statistic was compared against a null distribution of the said ratio, computed by sampling (*n* = 1000) the same number of sequences but labels of the groupings were randomly assigned. This comparison yielded an empirical *P* value. Bootstrapped (*n* = 1000) 95% confidence intervals were taken as error bars.

For the analyses of repertoire features, we collapsed each dataset to unique sequences (that is, for completely identical variable domain sequences, only 1 copy was kept) to reduce redundancies and inflation of sequence counts due to e.g., plasma cells being sampled during library preparation. We kept sequences within the same clonotype in non-singleton clones such for a more faithful representation of the overall makeup of the infiltrated B cell repertoire (so as to down-weigh singleton clonotypes which would otherwise distort the analysis if data collapsing was done on the clonotype level).

Lineage trees were generated with BrepPhylo (https://github.com/Fraternalilab/BrepPhylo) using the PHYLIP algorithm. Visualization and additional annotation were performed using ETE Toolkit 3^84^.

Manually generated lineage trees were generated by analyzing the sequences with IMGT/V-QUEST and representing the mutations relative to the unknown progenitor or the germline.

Healthy volunteers, Ebola patients and COVID-19 patients’ sequences were obtained from publicly available dataset produced with PacBio Technologies^37^. High-throughput dataset of tonsillar B cells was obtained from publicly available dataset using Illumina sequencing^40^. Sequences were annotated as described above, and a combination of V_gapped sequence and immunoglobulin heavy chain were used to investigate clonal expansion and mutational load. Quality control, data cleaning and removal of multiplicated UMIs were carried out as previously published. Immunoglobulin V-D-J gene usage and CDRH3 were determined using IMGT/High V-quest. Clonotype clustering was conducted as in previously published^37,85,86^. In brief, a Levenshtein distance matrix was generated on the CDRH3, hierarchically clustered and branches cut at 0.05 to generate clones.

For spatial transcriptomics analysis, single-cell RNA-seq (scRNA-seq) data of treatment-naive melanoma patients from GSE123139^87^, was used to deconvolute cell populations per spot in spatial transcriptomics (ST) data of in-house sourced melanoma tumors. The original cell annotation was used as reference during the integration between scRNA-seq and ST following Seurat pipeline (functions FindTransferAnchors and TransferData with default parameters). The publicly available, annotated, single-cell RNA-seq B cell data from King et al.^40^ provided as HumanTonsil_Bcells Seurat object, was used to identify presence of germinal centers in spatial transcriptomics (ST) data of melanoma tumors. Differentially expressed genes (DEGs) the annotated B cell populations were calculated using Seurat function FindAllMarkers with default parameters. The list of DEGs for germinal center (GC) B cells was filtered to include genes with adjusted *P* value ≤ 0.05, to create GC module. Subsequently, Seurat function AddModuleScore (default parameters) was used to calculate the average expression of the GC module per ST slice. R package ggvenn (version 0.1.9) was used create Venn diagram, between B cell/T cell deconvolution prediction score upper 50th percentile and GC prediction score upper 85th percentile.

### Bioinformatics evaluation of receptor revision

Ig sequences were analyzed using IMGT/V-QUEST^80,81^ to identify the V(D)J germlines, eventual mutations and CDR3. To evaluate VH replacement events, heavy chain sequences with the same CDR-H3 but different IGHV gene were aligned using the Needleman-Wunsch algorithm implemented in the EMBOSS Needle Pairwise Sequence Alignment tool^88^. Possible heptamers involved in the recombination were identified according to Itoh et al.^89^. To evaluate the VH replacement genealogical progression we annotated the genes with the position in the heavy chain locus according to IMGT website.

### Patient-derived recombinant antibody cloning and production

Matched and productive heavy and light chain variable regions, obtained as described above, were cloned into pVITRO1-IgG1/k or pVITRO1-IgG1/λ using the Polymerase Incomplete Primer Extension (PIPE) technique as previously described^79,89^. Briefly, pVITRO1-IgG1/k or pVITRO1-IgG1/λ and pCR-Blunt-VH and pCR-Blunt-VL were used as template PIPE PCR reactions to amplify the vector and the variable regions. Successfully cloned plasmid DNA was purified as described above.

Patient-derived recombinant IgG1 antibodies were produced as described before^90^. Briefly, pVITRO1 plasmids described above were used to transfect Expi293F cells using Expi293 Expression System Kit and recombinant IgG1 were purified from cell-culture supernatant by affinity chromatography using Protein A Columns (Pierce), according to manufacturer’s instructions.

### Immuno-mass spectrometry

Patient-derived recombinantly-expressed IgG1 (*n* = 12) antibodies were tested for their reactivity against tissue lysates from fresh frozen skin (*n* = 3) and melanoma (*n* = 2), alongside a negative (anti-NIP IgG1) and positive (anti-CSPG4 IgG1) control. Recombinant IgG1 were loaded on protein G magnetic beads and incubated with tissue lysates. The beads were then washed and the immunoprecipitated proteins were digested with Trypsin and analyzed by Liquid chromatography coupled to tandem mass spectrometry (LC-MS/MS) followed by Parallel reaction monitoring mass spectrometry (PRM-MS). Details regarding experimental and bioinformatic procedure are included the Supplementary Materials and Methods. The obtained list of proteins was filtered by removing: Ig heavy and light chains and protein contaminants (keratins, complement, apolipoproteins); proteins identified in the anti-NIP negative control; and proteins identified in only one replicate. For serum immuno-mass spectrometry we used serum samples from melanoma patients (*n* = 33) and healthy volunteers (*n* = 3) and tested IgG autoantibodies reactivity against a pool of 46 normal human tissues (from which were extracted more than 13000 antigens) as described in ref. ^91^.

### Statistical analyses

Statistical analyses of flow cytometric studies of B cell subsets were performed using GraphPad Prism software (version 9, GraphPad). Unpaired data are shown as truncated violin plots, median is represented with a thick dashed line, quartiles are represented with a thin dotted line. Analyses of matched tumor and blood samples are represented with paired dot plots, with lines connecting the pairs. Statistical significance was calculated with Mann–Whitney test (unpaired data) or Wilcoxon test (paired data), *P* values less than 0.05 were considered statistically significant. Single, double, triple, and quadruple asterisks indicate *P* < 0.05, *P* < 0.01, *P* < 0.001 and *P* < 0.0001, respectively. R version 4.1.2 and python version 3.9.7 were used to analyze B cell antibody repertoires. As regards, B cell antibody repertoire statistical analyses, for comparison of gene usage frequencies we used a two-sample proportion *z*-test, while for comparison of ratios of antibody class/subclass across different data groupings, the observed statistic was compared against a null distribution of the said ratio, as described in detail above (see “Antibody repertoire analysis”). Comparison of gene expression and antibody mutation rates was performed using Mann–Whitney test (GraphPad Prism software). Serum immuno-mass spectrometry presence absence clustering was performed using the Rvenn package in R.

### Reporting summary

Further information on research design is available in the Nature Portfolio Reporting Summary linked to this article.

## Supplementary information

Supplementary Information

Reporting Summary

## Data Availability

The data generated in this study are provided in the Supplementary Information/Source data file. Source data are provided with this paper.

## Source data

Source Data

## Code availability

The code used in this study have been deposited in the following github repository: https://github.com/josef0731/melanoma-ig.

## References

[1] Borst J, Ahrends T, Babala N, Melief CJM, Kastenmuller W (2018). CD4(+) T cell help in cancer immunology and immunotherapy. Nat. Rev. Immunol..

[2] Wei SC (2017). Distinct cellular mechanisms underlie anti-CTLA-4 and anti-PD-1 checkpoint blockade. Cell.

[3] Fridman WH, Zitvogel L, Sautes-Fridman C, Kroemer G (2017). The immune contexture in cancer prognosis and treatment. Nat. Rev. Clin. Oncol..

[4] Cabrita R (2020). Tertiary lymphoid structures improve immunotherapy and survival in melanoma. Nature.

[5] Griss J (2019). B cells sustain inflammation and predict response to immune checkpoint blockade in human melanoma. Nat. Commun..

[6] Willsmore ZN (2020). B cells in patients with melanoma: implications for treatment with checkpoint inhibitor antibodies. Front. Immunol..

[7] Chiaruttini G (2017). B cells and the humoral response in melanoma: the overlooked players of the tumor microenvironment. Oncoimmunology.

[8] Saul L (2016). IgG subclass switching and clonal expansion in cutaneous melanoma and normal skin. Sci. Rep..

[9] Erdag G (2012). Immunotype and immunohistologic characteristics of tumor-infiltrating immune cells are associated with clinical outcome in metastatic melanoma. Cancer Res..

[10] Garg K (2016). Tumor-associated B cells in cutaneous primary melanoma and improved clinical outcome. Hum. Pathol..

[11] Karagiannis, P. et al. Innate stimulation of B cells ex vivo enhances antibody secretion and identifies tumour-reactive antibodies from cancer patients. *Clin. Exp. Immunol.* **207**, 84–94 (2022). 10.1093/cei/uxab005 PMC8802180 35020866

[12] Ladanyi A (2011). Prognostic impact of B-cell density in cutaneous melanoma. Cancer Immunol. Immunother..

[13] Karagiannis P (2015). Elevated IgG4 in patient circulation is associated with the risk of disease progression in melanoma. Oncoimmunology.

[14] Diem S (2019). Immunoglobulin G and subclasses as potential biomarkers in metastatic melanoma patients starting checkpoint inhibitor treatment. J. Immunother..

[15] Karagiannis, P. et al. IgG4 subclass antibodies impair antitumor immunity in melanoma. *J. Clin. Investig.* **123**, 1457–1474 (2013). 10.1172/JCI65579 PMC3613918 23454746

[16] Bolotin DA (2017). Antigen receptor repertoire profiling from RNA-seq data. Nat. Biotechnol..

[17] Mose LE (2016). Assembly-based inference of B-cell receptor repertoires from short read RNA sequencing data with V’DJer. Bioinformatics.

[18] Evans RL, Pottala JV, Nagata S, Egland KA (2018). Longitudinal autoantibody responses against tumor-associated antigens decrease in breast cancer patients according to treatment modality. BMC Cancer.

[19] Wang S (2018). Using a panel of multiple tumor-associated antigens to enhance autoantibody detection for immunodiagnosis of gastric cancer. Oncoimmunology.

[20] Koziol JA, Imai H, Dai L, Zhang JY, Tan EM (2018). Early detection of hepatocellular carcinoma using autoantibody profiles from a panel of tumor-associated antigens. Cancer Immunol. Immunother..

[21] Li P (2017). Evaluation of serum autoantibodies against tumor-associated antigens as biomarkers in lung cancer. Tumour Biol..

[22] Sun H (2017). Serum autoantibodies against a panel of 15 tumor-associated antigens in the detection of ovarian cancer. Tumour Biol..

[23] Wu X (2017). Combined anti-VEGF and anti-CTLA-4 therapy elicits humoral immunity to Galectin-1 which is associated with favorable clinical outcomes. Cancer Immunol. Res..

[24] Michels J (2018). Multiplex bead-based measurement of humoral immune responses against tumor-associated antigens in stage II melanoma patients of the EORTC18961 trial. Oncoimmunology.

[25] Fässler M (2019). Antibodies as biomarker candidates for response and survival to checkpoint inhibitors in melanoma patients. J. Immunother. Cancer.

[26] Stockert E (1998). A survey of the humoral immune response of cancer patients to a panel of human tumor antigens. J. Exp. Med..

[27] Nagata Y (2002). Differential presentation of a soluble exogenous tumor antigen, NY-ESO-1, by distinct human dendritic cell populations. Proc. Natl Acad. Sci. USA.

[28] Sanz, I. et al. Challenges and opportunities for consistent classification of human B cell and plasma cell populations. *Front. Immunol*. **10**, 2458 (2019). 10.3389/fimmu.2019.02458 PMC6813733 31681331

[29] Nowicka M (2017). CyTOF workflow: differential discovery in high-throughput high-dimensional cytometry datasets. F1000Res.

[30] Egbuniwe, I. et al. B lymphocytes accumulate and proliferate in human skin at sites of cutaneous antigen challenge. *J. Invest. Dermatol.* **142**, 726–731 (2022). 10.1016/j.jid.2021.06.038 PMC8880055 34450137

[31] Li H (2020). Dysfunctional CD8 T cells form a proliferative, dynamically regulated compartment within human melanoma. Cell.

[32] Benito C, Gomis R, Fernández-Alvarez J, Usac EF, Gallart T (2003). Transcript expression of two Iglambda rearrangements and RAG-1/RAG-2 in a mature human B cell producing IgMlambda islet cell autoantibody. J. Clin. Immunol..

[33] Itoh K (2000). Immunoglobulin heavy chain variable region gene replacement as a mechanism for receptor revision in rheumatoid arthritis synovial tissue B lymphocytes. J. Exp. Med..

[34] Meng, W. et al. Trials and Tribulations with VH Replacement. *Front Immunol.* **5**, 10 (2014). 10.3389/fimmu.2014.00010 PMC3906580 24523721

[35] Prak ETL, Monestier M, Eisenberg RA (2011). B cell receptor editing in tolerance and autoimmunity. Ann. NY Acad. Sci..

[36] Nemazee D, Weigert M (2000). Revising B cell receptors. J. Exp. Med..

[37] Stewart, A. et al. Pandemic, epidemic, endemic: b cell repertoire analysis reveals unique anti-viral responses to SARS-CoV-2, ebola and respiratory syncytial virus. *Front. Immunol.* **13**, 807104 (2022). 10.3389/fimmu.2022.807104 PMC9111746 35592326

[38] Townsend CL (2016). Significant differences in physicochemical properties of human immunoglobulin kappa and lambda CDR3 regions. Front. Immunol..

[39] Mallaby, J. et al. Diversification of immunoglobulin genes by gene conversion in the domestic chicken (*Gallus gallus domesticus*). *Discov. Immunol.* **2**, kyad002 (2023). 10.1093/discim/kyad002 PMC10917233 38567069

[40] King, H. W. et al. Single-cell analysis of human B cell maturation predicts how antibody class switching shapes selection dynamics. *Sci. Immunol*. **6**, eabe6291 (2021). 10.1126/sciimmunol.abe6291 33579751

[41] Laffy JMJ (2017). Promiscuous antibodies characterised by their physico-chemical properties: from sequence to structure and back. Prog. Biophys. Mol. Biol..

[42] Wilson, P. C. et al. Receptor revision of immunoglobulin heavy chain variable region genes in normal human B lymphocytes. *J. Exp. Med*. **191**, 1881–1894 (2000). 10.1084/jem.191.11.1881 PMC2213516 10839804

[43] Edwards MR (2002). Analysis of IgE antibodies from a patient with atopic dermatitis: biased V gene usage and evidence for polyreactive IgE heavy chain complementarity-determining region 3. J. Immunol..

[44] Bashford-Rogers RJM, Smith KGC, Thomas DC (2018). Antibody repertoire analysis in polygenic autoimmune diseases. Immunology.

[45] Lim B (2019). Putative autoantibodies in the cerebrospinal fluid of Alzheimer’s disease patients. F1000Res.

[46] Music M (2019). A proteome-wide immuno-mass spectrometric identification of serum autoantibodies. Clin. Proteomics.

[47] Music M (2019). Correction to: A proteome-wide immuno-mass spectrometric identification of serum autoantibodies. Clin. Proteomics.

[48] Cheng KC (2015). Proteomic surveillance of putative new autoantigens in thyroid orbitopathy. Br. J. Ophthalmol..

[49] Ruchala M (2007). The prevalence of autoantibodies to: myosin, troponin, tropomyosin and myoglobin in patients with circulating triiodothyronine and thyroxine autoantibodies (THAA). Neuro Endocrinol. Lett..

[50] Shi LL (2015). Prohibitin as a novel autoantigen in rheumatoid arthritis. Cent. Eur. J. Immunol..

[51] Wang JY, Zhang W, Rho JH, Roehrl MW, Roehrl MH (2020). A proteomic repertoire of autoantigens identified from the classic autoantibody clinical test substrate HEp-2 cells. Clin. Proteomics.

[52] Du H (2015). Prohibitin is involved in patients with IgG4 related disease. PLoS ONE.

[53] Dai L (2017). Identification of autoantibodies to ECH1 and HNRNPA2B1 as potential biomarkers in the early detection of lung cancer. Oncoimmunology.

[54] Kondo M (2011). Identification of autoantibodies against TRPM1 in patients with paraneoplastic retinopathy associated with ON bipolar cell dysfunction. PLoS ONE.

[55] Lacombe J (2013). Identification and validation of new autoantibodies for the diagnosis of DCIS and node negative early-stage breast cancers. Int. J. Cancer.

[56] Suzuki A (2010). Identification of melanoma antigens using a Serological Proteome Approach (SERPA). Cancer Genomics Proteomics.

[57] Harris RJ (2021). Tumor-infiltrating B lymphocyte profiling identifies IgG-biased, clonally expanded prognostic phenotypes in triple-negative breast cancer. Cancer Res..

[58] Cipponi A (2012). Neogenesis of lymphoid structures and antibody responses occur in human melanoma metastases. Cancer Res..

[59] Lua W-H (2018). The effects of antibody engineering CH and CL in Trastuzumab and Pertuzumab recombinant models: Impact on antibody production and antigen-binding. Sci. Rep..

[60] Bashford-Rogers, R. J. M. et al. Antibody repertoire analysis in polygenic autoimmune diseases. *Immunology* **155**, 3–17 (2018). 10.1111/imm.12927 PMC6099162 29574826

[61] Guthmiller JJ (2020). Polyreactive broadly neutralizing B cells are selected to provide defense against pandemic threat influenza viruses. Immunity.

[62] Hillion S, Rochas C, Youinou P, Jamin C (2005). Expression and reexpression of recombination activating genes: relevance to the development of autoimmune states. Ann. NY Acad. Sci..

[63] Dieker J (2016). Autoantibodies against modified histone peptides in SLE patients are associated with disease activity and lupus nephritis. PLoS ONE.

[64] Rho JH, Lampe PD (2013). High-throughput screening for native autoantigen-autoantibody complexes using antibody microarrays. J. Proteome Res..

[65] Zaenker P (2018). A diagnostic autoantibody signature for primary cutaneous melanoma. Oncotarget.

[66] Cohen BE, Manga P, Lin K, Elbuluk N (2020). Vitiligo and melanoma-associated vitiligo: understanding their similarities and differences. Am. J. Clin. Dermatol..

[67] Bhattacharya T (2016). Co-existence of psoriasis and melanoma in a large urban academic centre population: a cross-sectional retrospective study. J. Eur. Acad. Dermatol. Venereol..

[68] Monroy-Iglesias, M. J. et al. Antibodies as biomarkers for cancer risk: a systematic review. *Clin. Exp. Immunol.* **209**, 46–63 (2022). 10.1093/cei/uxac030 PMC9307228 35380164

[69] Liyanage UE (2022). Multi-trait genetic analysis identifies autoimmune loci associated with cutaneous melanoma. J. Invest. Dermatol..

[70] Motofei IG (2019). Melanoma and autoimmunity: spontaneous regressions as a possible model for new therapeutic approaches. Melanoma Res..

[71] Teulings HE (2015). Vitiligo-like depigmentation in patients with stage III-IV melanoma receiving immunotherapy and its association with survival: a systematic review and meta-analysis. J. Clin. Oncol..

[72] Zitvogel L, Perreault C, Finn OJ, Kroemer G (2021). Beneficial autoimmunity improves cancer prognosis. Nat. Rev. Clin. Oncol..

[73] Balch CM (2009). Final version of 2009 AJCC melanoma staging and classification. J. Clin. Oncol..

[74] Gershenwald JE, Scolyer RA (2018). Melanoma Staging: American Joint Committee on Cancer (AJCC) 8th Edition and beyond. Ann. Surg. Oncol..

[75] McInnes, L., Healy, J., Saul, N. & Großberger, L. UMAP: uniform manifold approximation and projection. *J. Open Source Softw*. **3**, 861 (2018).

[76] Levine JH (2015). Data-driven phenotypic dissection of AML reveals progenitor-like cells that correlate with prognosis. Cell.

[77] Hao Y (2021). Integrated analysis of multimodal single-cell data. Cell.

[78] James LK (2012). Allergen specificity of IgG(4)-expressing B cells in patients with grass pollen allergy undergoing immunotherapy. J. Allergy Clin. Immunol..

[79] Correa I (2018). Evaluation of antigen-conjugated fluorescent beads to identify antigen-specific B cells. Front. Immunol..

[80] Brochet X, Lefranc M-P, Giudicelli V (2008). IMGT/V-QUEST: the highly customized and integrated system for IG and TR standardized V-J and V-D-J sequence analysis. Nucleic Acids Res..

[81] Giudicelli V, Brochet X, Lefranc M-P (2011). IMGT/V-QUEST: IMGT standardized analysis of the immunoglobulin (IG) and T cell receptor (TR) nucleotide sequences. Cold Spring Harb. Protoc..

[82] Margreitter C (2018). BRepertoire: a user-friendly web server for analysing antibody repertoire data. Nucleic Acids Res..

[83] Aouinti S, Malouche D, Giudicelli V, Kossida S, Lefranc M-P (2015). IMGT/HighV-QUEST statistical significance of IMGT clonotype (AA) diversity per gene for standardized comparisons of next generation sequencing immunoprofiles of immunoglobulins and T cell receptors. PLoS ONE.

[84] Huerta-Cepas J, Serra F, Bork P (2016). ETE 3: reconstruction, analysis, and visualization of phylogenomic data. Mol. Biol. Evol..

[85] Wu YC (2010). High-throughput immunoglobulin repertoire analysis distinguishes between human IgM memory and switched memory B-cell populations. Blood.

[86] Martin, V. et al. Age‐related aspects of human IgM^+^ B cell heterogeneity. *Ann. NY Acad. Sci*. **1362**, 153–163 (2015). 10.1111/nyas.12823 PMC4758400 26152370

[87] Li H (2019). Dysfunctional CD8 T cells form a proliferative, dynamically regulated compartment within human melanoma. Cell.

[88] Madeira F (2019). The EMBL-EBI search and sequence analysis tools APIs in 2019. Nucleic Acids Res..

[89] Dodev TS (2014). A tool kit for rapid cloning and expression of recombinant antibodies. Sci. Rep..

[90] Ilieva KM (2017). Functionally active Fc mutant antibodies recognizing cancer antigens generated rapidly at high yields. Front. Immunol..

[91] Di Meo, A. et al. Proteomic profiling of the human tissue and biological fluid proteome. *J. Proteome Res.* **20**, 444–452 (2021). 10.1021/acs.jproteome.0c00502 33107741

