# Supplementary material for: B cell profiles, antibody repertoire and reactivity reveal dysregulated responses with autoimmune features in melanoma
Source: Nat Commun. 2023 Jun 8;14:3378. doi: 10.1038/s41467-023-39042-y (PMC10249578; doi:10.1038/s41467-023-39042-y)
Supplement: Supplementary file 1 — Supplementary Information [file 41467_2023_39042_MOESM1_ESM.pdf]

# B cell profiles, antibody repertoire and reactivity reveal dysregulated responses with autoimmune features in melanoma

**Authors:** Silvia Crescioli[1], Isabel Correa[1], Joseph Ng[2,3], Zena N. Willsmore[1], Roman Laddach[1,4], Alicia Chenoweth[1,5], Jitesh Chauhan[1], Ashley Di Meo[6], Alexander Stewart[7], Eleni Kalliolia[1], Elena Alberts[5], Rebecca Adams[1], Robert J. Harris[1], Silvia Mele[1], Giulia Pellizzari[1], Anna B. M. Black[1], Heather J. Bax[1], Anthony Cheung[1,5], Mano Nakamura[1], Ricarda M. Hoffmann[1], Manuela Terranova-Barberio[1], Niwa Ali[8,9], Ihor Batruch[6], Antoninus Soosaipillai[6], Ioannis Prassas[6], Antigona Ulndreaj[6], Miyo K. Chatanaka[6,10], Rosamund Nuamah[11], Shichina Kannambath[11,12], Pawan Dhami[11], Jenny L. C. Geh[13,14], Alastair D. MacKenzie Ross[14], Ciaran Healy[14], Anita Grigoriadis[5], David Kipling[7], Panagiotis Karagiannis[1,15], Deborah K Dunn-Walters[7], Eleftherios P. Diamandis[6,10,16,17], Sophia Tsoka[4], James Spicer[18], Katie E. Lacy[1], Franca Fraternali[2,3], Sophia N. Karagiannis[1,5]*.

**Affiliations:**

[1] St John's Institute of Dermatology, School of Basic & Medical Biosciences, King's College London, Guy's Hospital; London, United Kingdom

[2] Randall Centre for Cell and Molecular Biophysics, King's College London; London, United Kingdom

[3] Research Department of Structural and Molecular Biology, University College London; London, United Kingdom

[4] Department of Informatics, Faculty of Natural, Mathematical and Engineering Sciences, King's College London; London, United Kingdom

[5] Breast Cancer Now Research Unit, School of Cancer & Pharmaceutical Sciences, King's College London, Guy's Hospital; London, United Kingdom

[6] Lunenfeld-Tanenbaum Research Institute, Mount Sinai Hospital; Toronto, Canada

[7] School of Biosciences and Medicine, University of Surrey; Guildford, United Kingdom

[8] Peter Gorer Department of Immunobiology, School of Immunology and Microbial Sciences, Faculty of Life Sciences and Medicine, King's College London, London, UK

[9] Centre for Gene Therapy and Regenerative Medicine, School of Basic and Medical Biosciences, Faculty of Life Sciences and Medicine, King's College London, London, UK

[10] University of Toronto, Department of Laboratory Medicine and Pathobiology; Toronto, Canada

[11] Biomedical Research Centre, Guy's and St. Thomas' NHS Foundation Trust; London, United Kingdom

[12] Genomics Facility, Institute of Cancer Research; London, United Kingdom

[13] St John's Institute of Dermatology, Guy's, King's, and St. Thomas' Hospitals NHS Foundation Trust; London, United Kingdom

[14] Department of Plastic Surgery at Guy's and St. Thomas' NHS Foundation Trust; London, United Kingdom

[15] Department of Cancer Biology, Dana-Farber Cancer Institute, Boston, MA, USA.

[16] Department of Pathology and Laboratory Medicine, Mount Sinai Hospital; Toronto, Canada

[17] Department of Clinical Biochemistry, University Health Network; Toronto, Canada

[18] School of Cancer & Pharmaceutical Sciences, King's College London, Guy's Hospital; London, United Kingdom

*Corresponding author: Sophia N. Karagiannis, E-mail: sophia.karagiannis@kcl.ac.uk

**Supplementary Information**

Supplementary Figure 1: Gating strategy and differential abundance analysis to compare healthy volunteers and melanoma patients.

Supplementary Figure 2: B cell infiltrate in cutaneous melanoma metastases.

Supplementary Figure 3: Antibody repertoire on Ig sequences isolated from melanoma patient's blood and tumor.

Supplementary Figure 4: VH replacement.

Supplementary Figure 5: *AICDA (AID)* gene expression in cutaneous samples.

Supplementary Figure 6: V gene distribution per isotype.

Supplementary Figure 7: Immuno-mass spectrometry via Shotgun Mass-Spectrometry LC-MS/MS.

Supplementary Figure 8: Glycan array heatmap 1.

Supplementary Figure 9: Glycan array heatmap 2.

Supplementary Figure 10: Serum immuno-mass spectrometry study pipeline.

Supplementary Figure 11: Heatmap of serum immuno-mass spectrometry (IMS) peak area.

Supplementary Figure 12: Gene expression of tubulins in human skin and melanoma lesions.

Supplementary Figure 13: Heatmaps of marker expression for B cells and T cells detected by CyTOF analyses in melanoma patient blood samples.

Supplementary Table 1: Criteria used for labelling and merging CyTOF clusters.

Supplementary Table 2: Number of sequences per sample in high throughput antibody repertoire from melanoma skin metastases.

Supplementary Table 3: Number of heavy chain sequences per cohort in high throughput antibody repertoire

Supplementary Table 4: Melanoma patients recruited for PBMC analysis by CyTOF.

Supplementary Table 5: Healthy volunteers recruited for PBMC analysis by CyTOF.

Supplementary Table 6: Melanoma patients recruited for PBMC analysis by Flow Cytometry.

Supplementary Table 7: Melanoma lesion specimens.

Supplementary Table 8: Melanoma patients recruited for serum immuno-mass spectrometry analysis.

Supplementary Table 9: CyTOF antibody panel.

Supplementary Methods

Supplementary References

**Supplementary Figure 1**

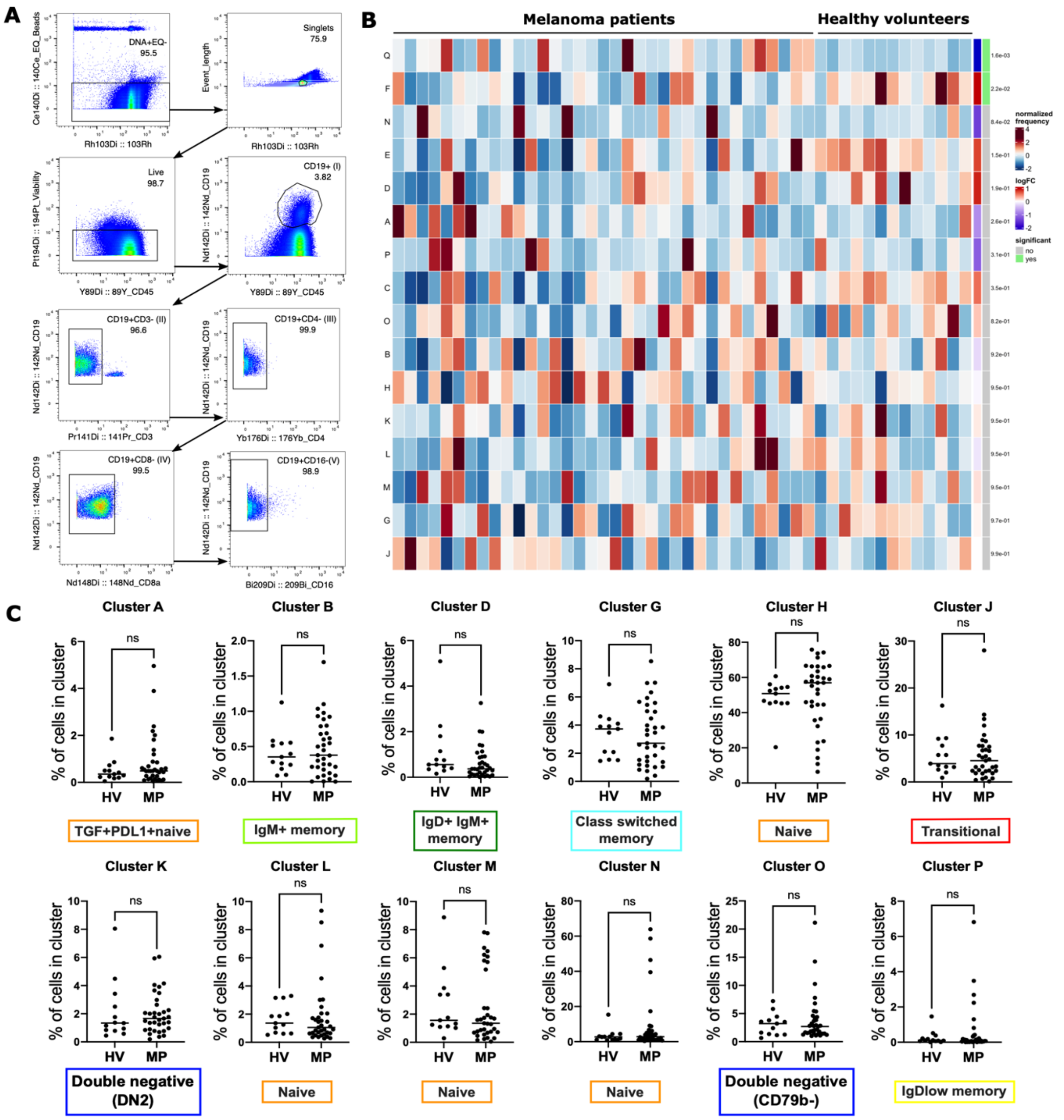


**Supplementary Figure 1: Gating strategy and differential abundance analysis to compare healthy volunteers and melanoma patients**. (**A**) CyTOF gating strategy to identify B cell subsets for analysis. (B-C) biologically independent samples: Healthy volunteers, n = 13; Melanoma patients, n = 35. (**B**) Differential Abundance analysis (DA test) comparing healthy volunteer and melanoma patient samples. (**C**) Scatter dot plot showing the non-significant comparisons of the relative abundance of B cell clusters in each sample between healthy

volunteers and melanoma patients. Statistical significance was calculated with the Mann-Whitney test.

**Supplementary Figure 2**

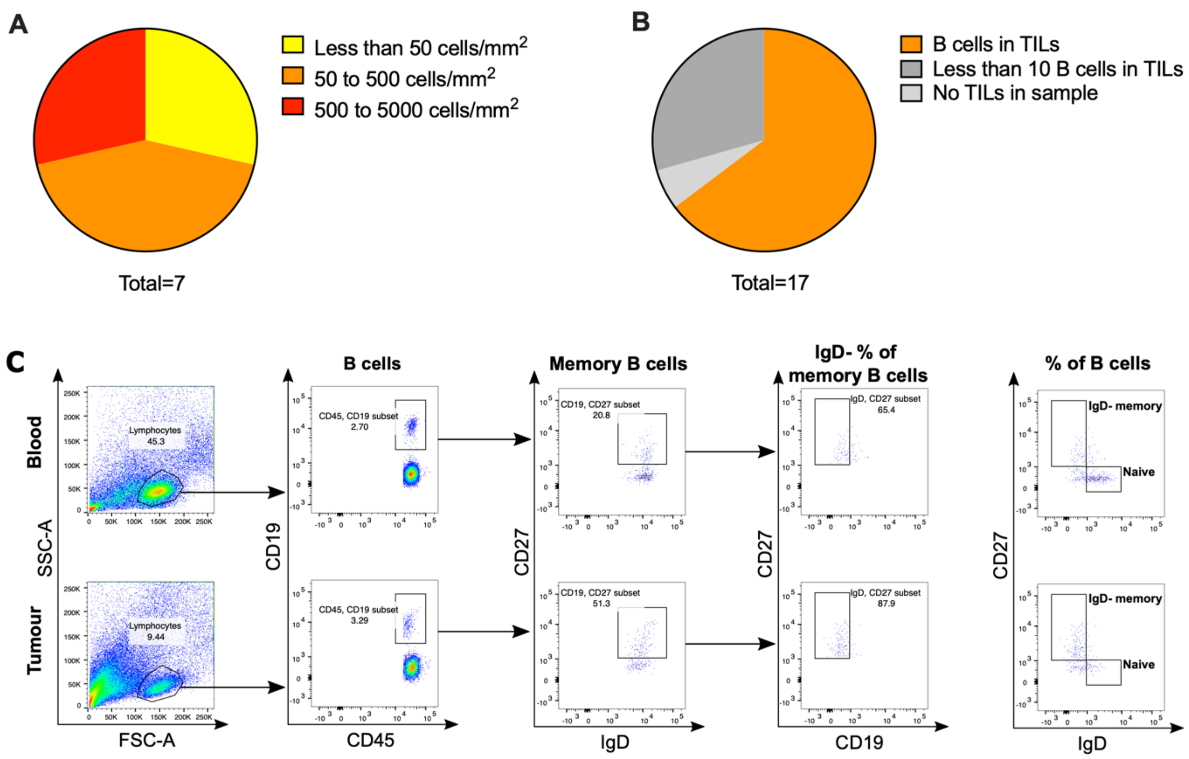


**Supplementary Figure 2: B cell infiltration in cutaneous melanoma metastases.** (**A**) Pie chart representing the presence and density of CD20+ B cells in tumor samples (intratumoral and peritumoral) analyzed by immunofluorescence (n = 7). The numbers of CD20+ B cells per mm$^2$ were calculated using QuPath software. (**B**) Pie chart representing the presence of B cells (CD19+ cells) in Tumor infiltrating Lymphocytes (TILs) (CD45+ cells) in the 17 tumor samples analyzed by flow cytometry. (**C**) Flow cytometry gating strategy for B cell phenotyping in melanoma patient blood and tumor samples.

## Supplementary Figure 3

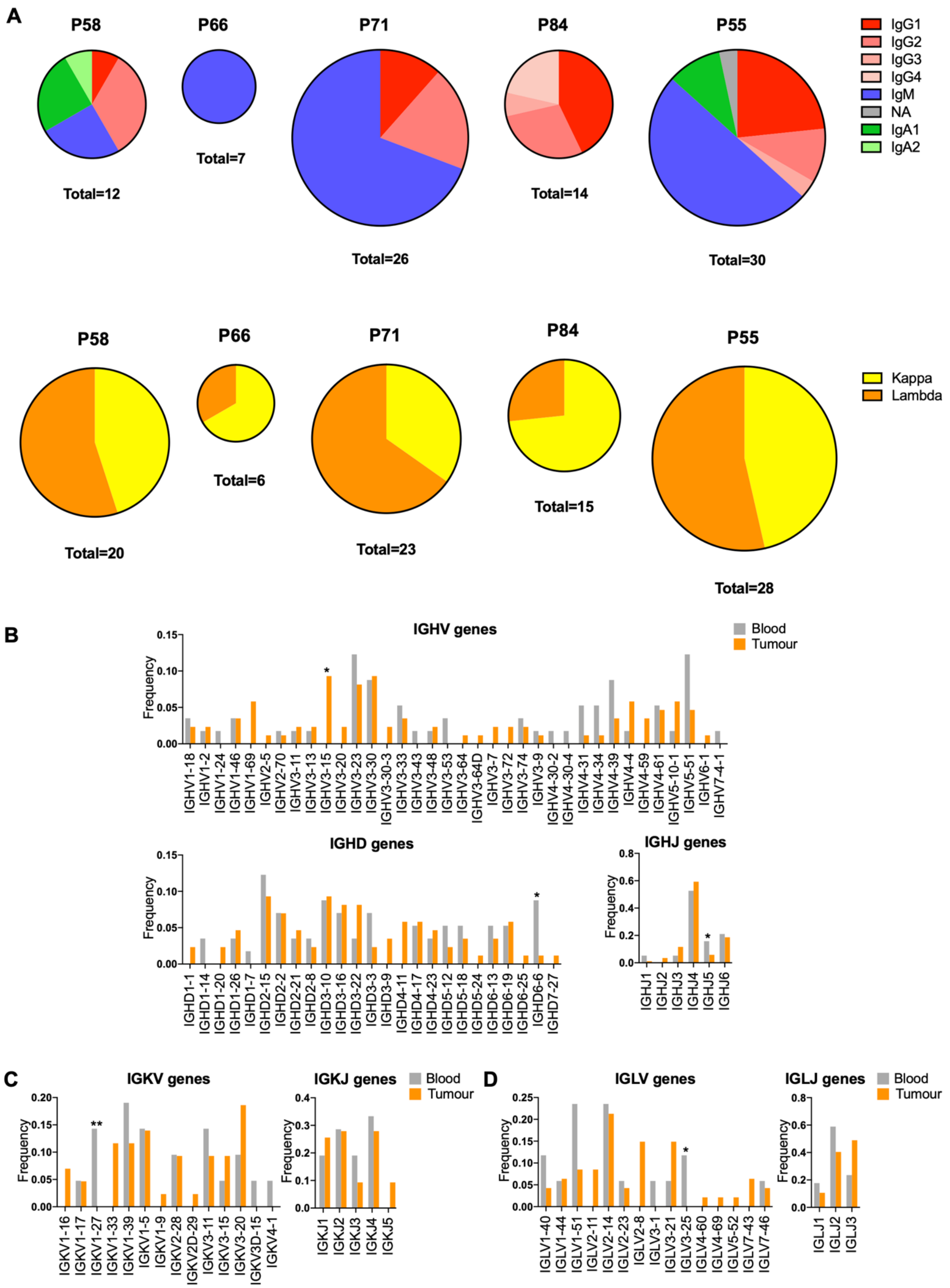

**Supplementary Figure 3: Antibody repertoire on IgD- memory B cells isolated from melanoma patient blood and tumor samples.** (**A**) Pie charts representing the isotype distribution per patient; the values below each chart depict the numbers of heavy chain sequences per patient. (**B-D**) Comparison between melanoma patients' blood (grey, n = 11; 57 heavy chain and 38 light chain sequences) and tumor (orange, n = 5; 89 heavy chain and 92 light chain sequences). (**B**) Frequency distribution of heavy chain IGHV, IGHD and IGHJ genes. (**C**) Frequency distribution of kappa light chain IGKV and IGKJ genes and (**D**) lambda light chain IGLV and IGLJ genes. Statistical significance was calculated with a two-sample proportion z-test; *P < 0.05, **P < 0.01.

**Supplementary Figure 4**

```
                         <-------------------------FR1-IMGT--------------------------
IGH-V4-4*01 germ         CAGGTGCAGCTGCAGGAGTCGGGCCCAGGACTGGTGAAGCCTCCGGGGACCCTGTCCCTC
P21_B12_1                CAGGTGCAGCTGCAGGAGTCGGGCCCAGGACTGGTGAAGCCTCCGGGGACCCTGTCCCTC
                              ************************************* *** *************
P21_B9_5                 -----GCAGCTGCAGGAGTCGGGCCCAGGACTGGTGAAGCCTTCGGAGACCCTGTCCCTC
IGH-V4-59*01 germ        CAGGTGCAGCTGCAGGAGTCGGGCCCAGGACTGGTGAAGCCTTCGGAGACCCTGTCCCTC

                         -------------->__________CDR1-IMGT________<-----------------
IGH-V4-4*01 germ         ACCTGCGCTGTCTCTGGTGGCTCCATCAGCAGTAGTAACTGGTGGAGTTGGGTCCGCCAG
P21_B12_1                GCCTGCGGTGTCTCTGGTGGGTCAGTCATCACTAGTCACTGGTGGACTTGGGTCCGCCAG
                          *****  ************ **   ***** ****  **  ****  *** **** ***
P21_B9_5                 ACCTGCACTGTCTCTGGTGGCTC---CATCAGTAGTTTCTACTGGAGCTGGATCCGGCAG
IGH-V4-59*01 germ        ACCTGCACTGTCTCTGGTGGCTC---CATCAGTAGTTACTACTGGAGCTGGATCCGGCAG

                         -----FR2-IMGT-------------------->______CDR2-IMGT_____<-----
IGH-V4-4*01 germ         CCCCCAGGGAAGGGGCTGGAGTGGATTGGGGAAATCTATCATAGTGGGAGCACCAACTAC
P21_B12_1                ACCCCAGGGAAGGGCCTGGAGTGGATTGGGGAAGTCTATCATACTGGGAGCACCAACTAC
                          ************* *************** *   ***** * * ****************
P21_B9_5                 CCCCCAGGGAAGGGACTGGAGTGGATTGGGTATTTCTATTACAGTGGGAGCACCAACTAC
IGH-V4-59*01 germ        CCCCCAGGGAAGGGACTGGAGTGGATTGGGTATATCTATTACAGTGGGAGCACCAACTAC

                         -----------------------------------FR3-IMGT-----------------
IGH-V4-4*01 germ         AACCCGTCCCTCAAGAGTCGAGTCACCATATCAGTAGACAAGTCCAAGAACCAGTTCTCC
P21_B12_1                AACCCGTCCCTCAAGAGTCGAGTCACCATGTCAGTGGACAAGTCCAGGAACCAGTTCTCC
                         ************************************************************
P21_B9_5                 AACCCGTCCCTCAAGAGTCGAGTCACCATGTCAGTGGACAAGTCCAGGAACCAGTTCTCC
IGH-V4-59*01 germ        AACCCCTCCCTCAAGAGTCGAGTCACCATATCAGTAGACACGTCCAAGAACCAGTTCTCC

                         ----------------------------------------------->___CDR3_____
IGH-V4-4*01 germ         CTGAAGCTGAGCTCTGTGACCGCCGCGGACACGGCCGTGTATTGCTGTGCGAGAG-----
P21_B12_1                CTGAAGTTGAGCTCTGTGACCGCCGCGGACACGGCCACATATTTTTGTGCGAGAGGAGAG
                         ********************************************************
P21_B9_5                 CTGAAGTTGAGCTCTGTGACCGCCGCGGACACGGCCACATATTTTTGTGCGAGAGGAGAG
IGH-V4-59*01 germ        CTGAAGCTGAGCTCTGTGACCGCCGCGGACACGGCCGTGTATTACTGTGCGAGAGA----
```

**Supplementary Figure 4: VH replacement.** cDNA VH sequence alignment of the example in Figure 5E (M847_IgG_B12_1 and M847_IgG_B9_5 clones and germlines) with Frameworks and CDRs annotation according to IMGT. Asterisks: identical nucleotides in clones; Purple font: identical nucleotides in clones and germline IGH-V4-4*01 only; Yellow font: identical nucleotides in M847_IgG_B12_1 and germline IGH-V4-4*01 only; Blue font: identical nucleotides in M847_IgG_B9_5 clone and germline IGH-V4-59*01 only; Yellow highlight: unique nucleotides in M847_IgG_B12_1 clone; Light blue highlight: unique nucleotides in M847_IgG_B9_5 clone; Green highlight: nucleotides in both clones but not in germlines; Red font: possible heptamer site for recombination.

**Supplementary Figure 5**

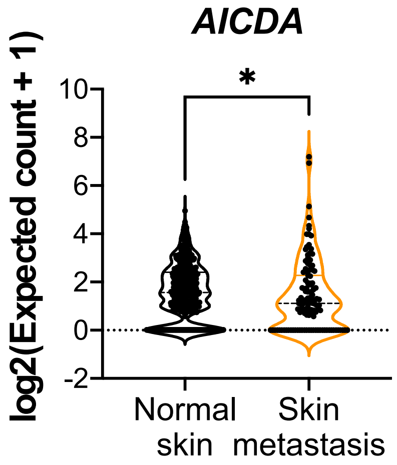


**Supplementary Figure 5: *AICDA (AID)* gene expression in cutaneous samples.** *AICDA (AID)*, gene expression in normal skin (n = 555) and melanoma skin metastases (n = 116) (RSEM expected count (DESeq2 standardized) dataset, TCGA TARGET GTEx study, UCSC Xena).

**Supplementary Figure 6**

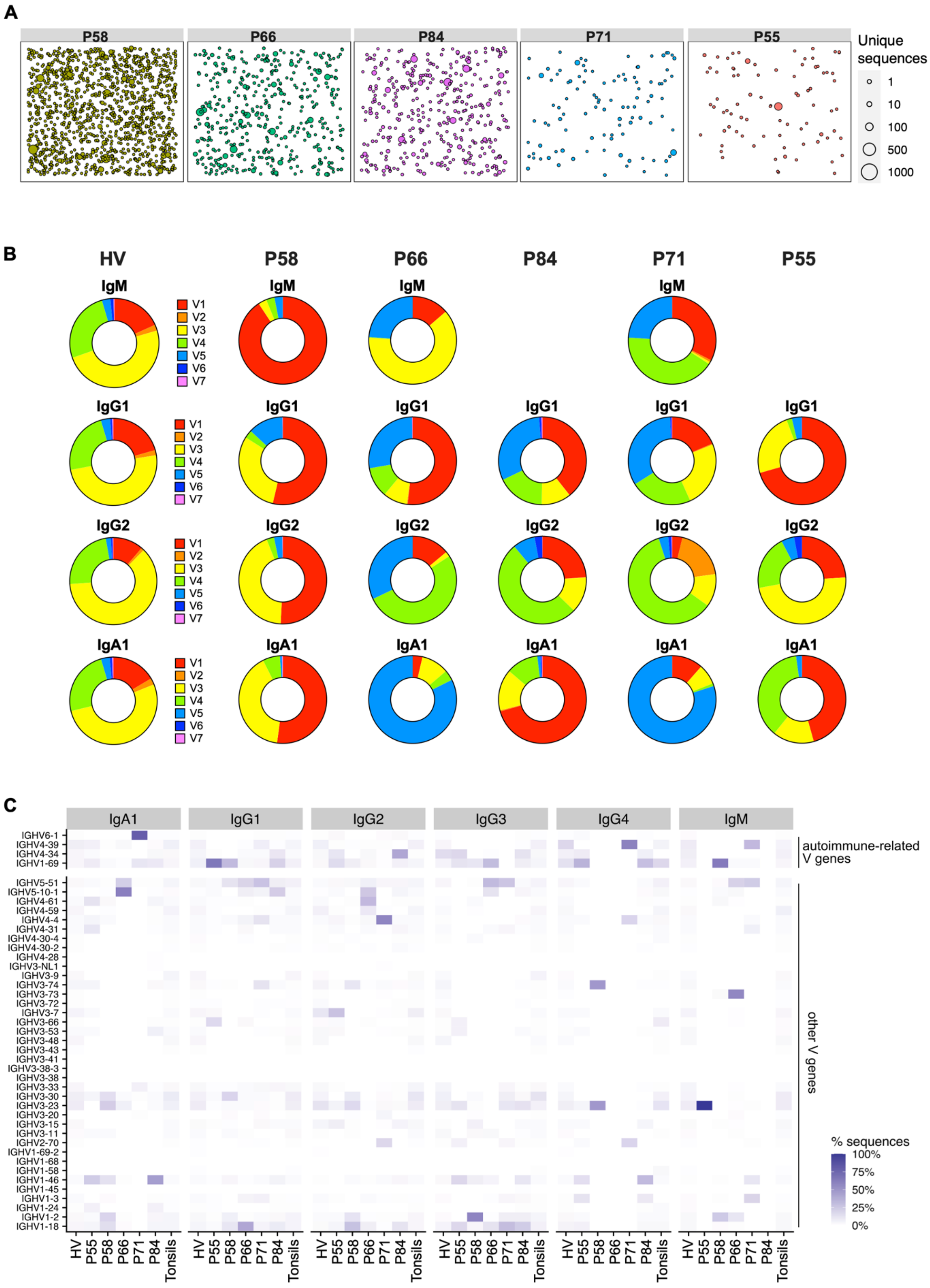

**Supplementary Figure 6: V gene distribution per antibody isotype.** (**A**) Representation of IgH clones and clonal expansion per tumor sample: the circle size is proportional to the number of unique sequences per clone. (**B**) Pie charts representing V family distribution in healthy volunteers (HV) and melanoma patients (MP) per isotype, divided per tumor sample. (**B**) Heatmap representing V gene proportions for each antibody isotype detected in each tumor sample in comparison with the average of healthy volunteer blood and tonsil samples V family distribution. The top 4 V genes have been associated with autoimmunity[1].

## Supplementary Figure 7

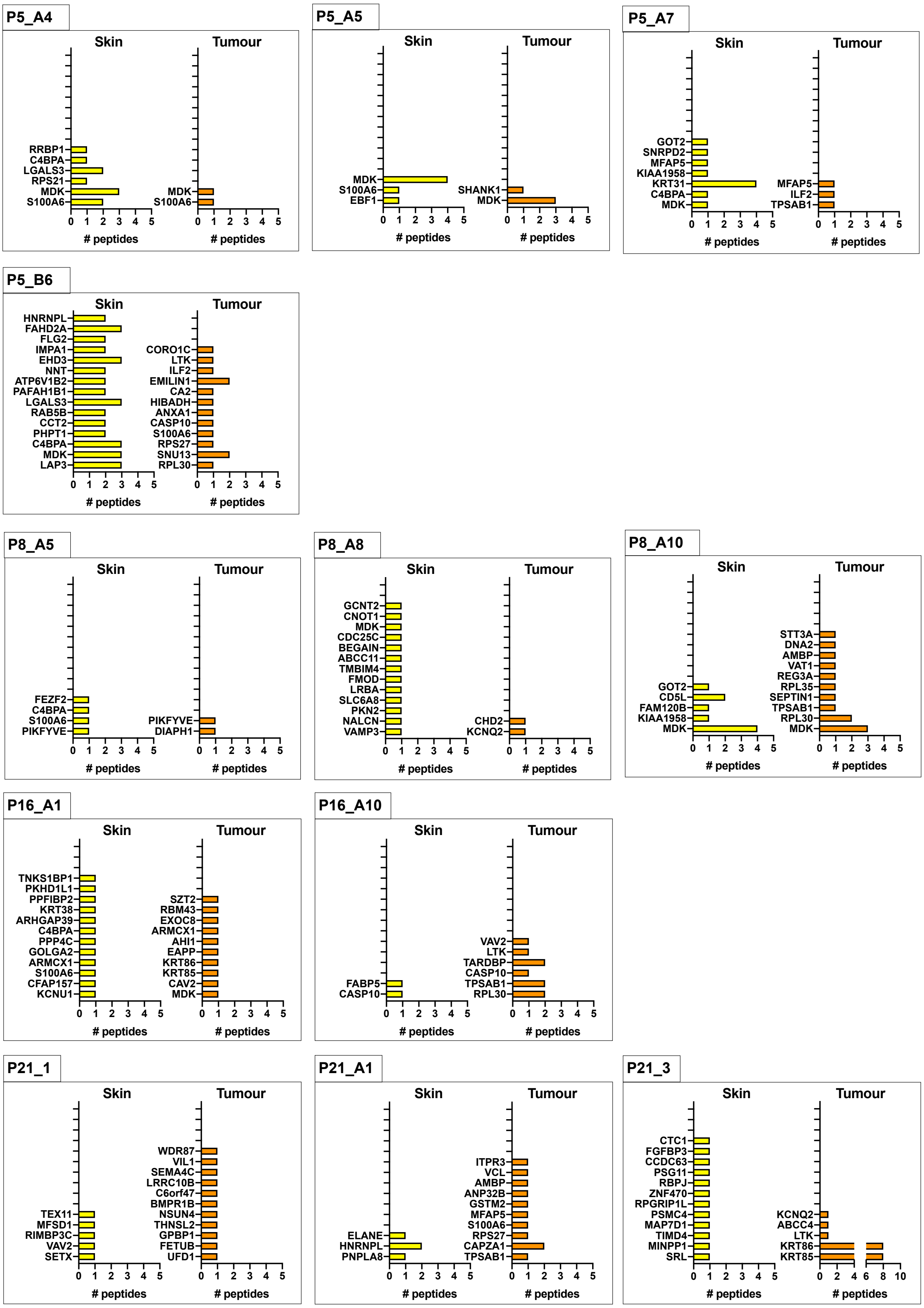

**Supplementary Figure 7: Immuno-mass spectrometry via Shotgun Mass-Spectrometry LC-MS/MS.** Bar charts representing, for every antibody, the proteins identified in the immuno-mass spectrometry via Shotgun Mass-Spectrometry LC-MS/MS in Normal Skin and Melanoma Tumor. In the histograms is shown the number of peptides identified for each protein.

## Supplementary Figure 8

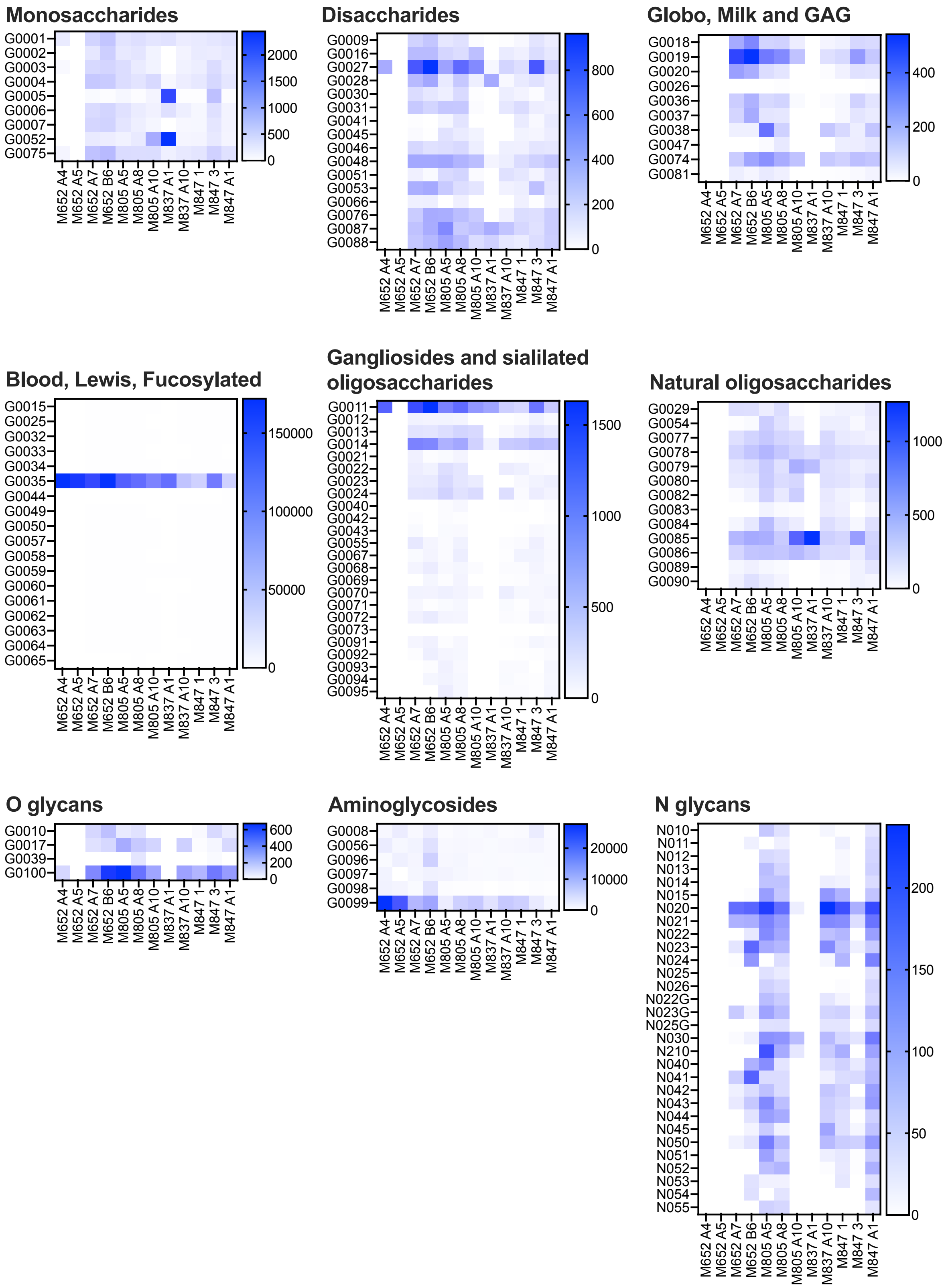

**Supplementary Figure 8: Glycan array heatmap 1.** Heatmap representing the analyzed glycan array results grouped the glycans classes according to Glycan Array 300 manufacturers: "Monosaccharides", "Disaccharides", "Globo, Milk and GAG", "Blood, Lewis, Fucosylated", "Gangliosides and sialylated oligosaccarides", "Natural oligosaccharides", "O glycans", "Aminoglycosides", "N glycans".

**Supplementary Figure 9**

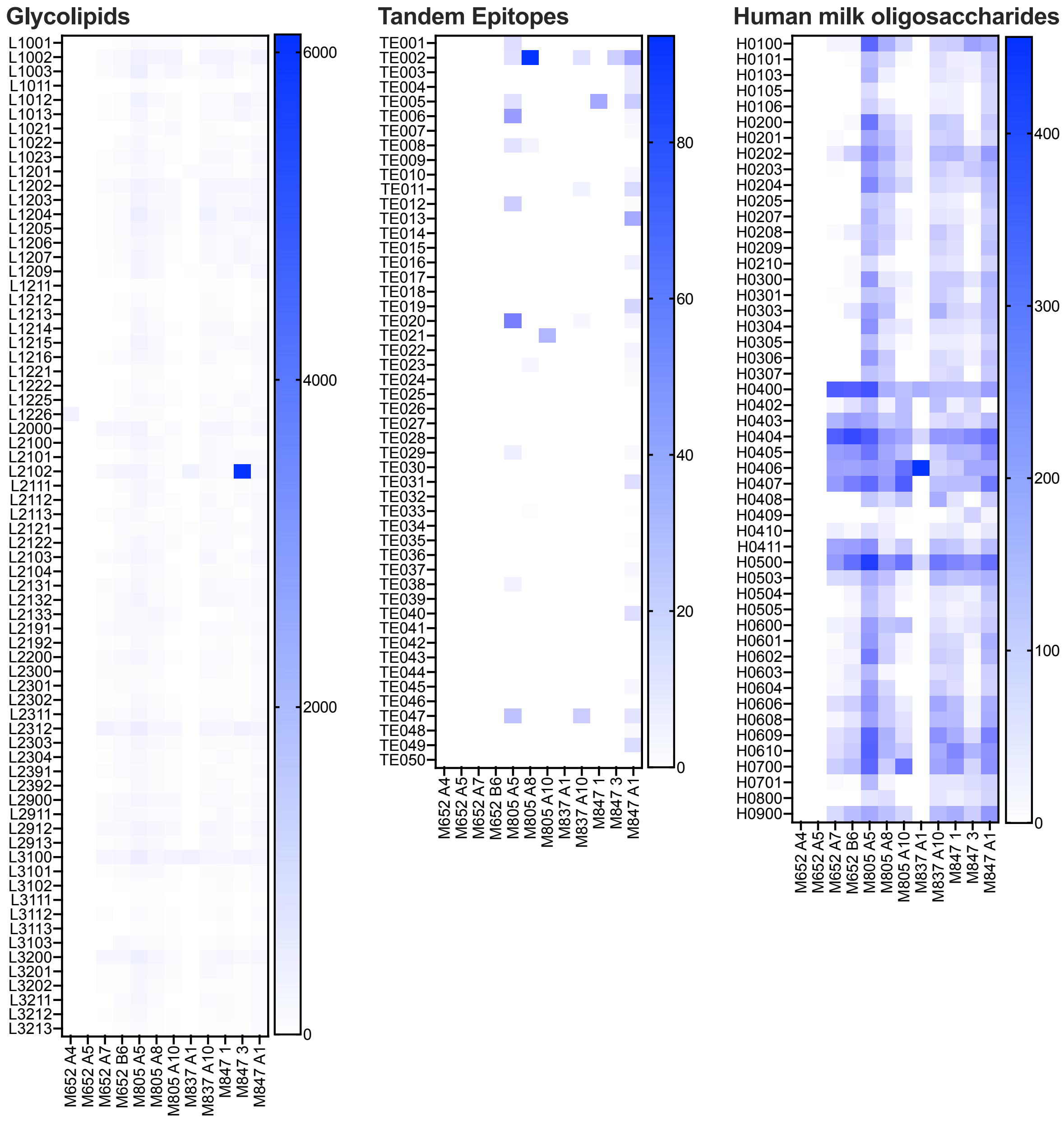


**Supplementary Figure 9: Glycan array heatmap 2.** Heatmap representing the analyzed glycan array results grouped the glycans classes according to Glycan Array 300 manufacturers: "Glycolipids", "Tandem Epitopes", "Human milk oligosaccharides".

**Supplementary Figure 10**

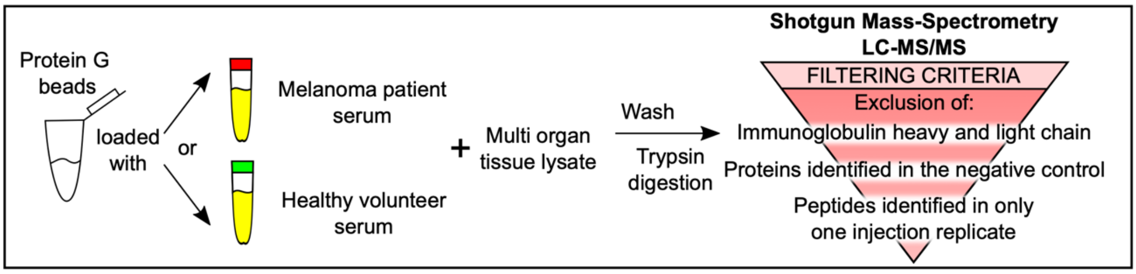


**Supplementary Figure 10: Serum immuno-mass spectrometry study pipeline.** Schematic of immuno-mass spectrometry workflow for patient and healthy volunteer serum sample analysis.

**Supplementary Figure 11**

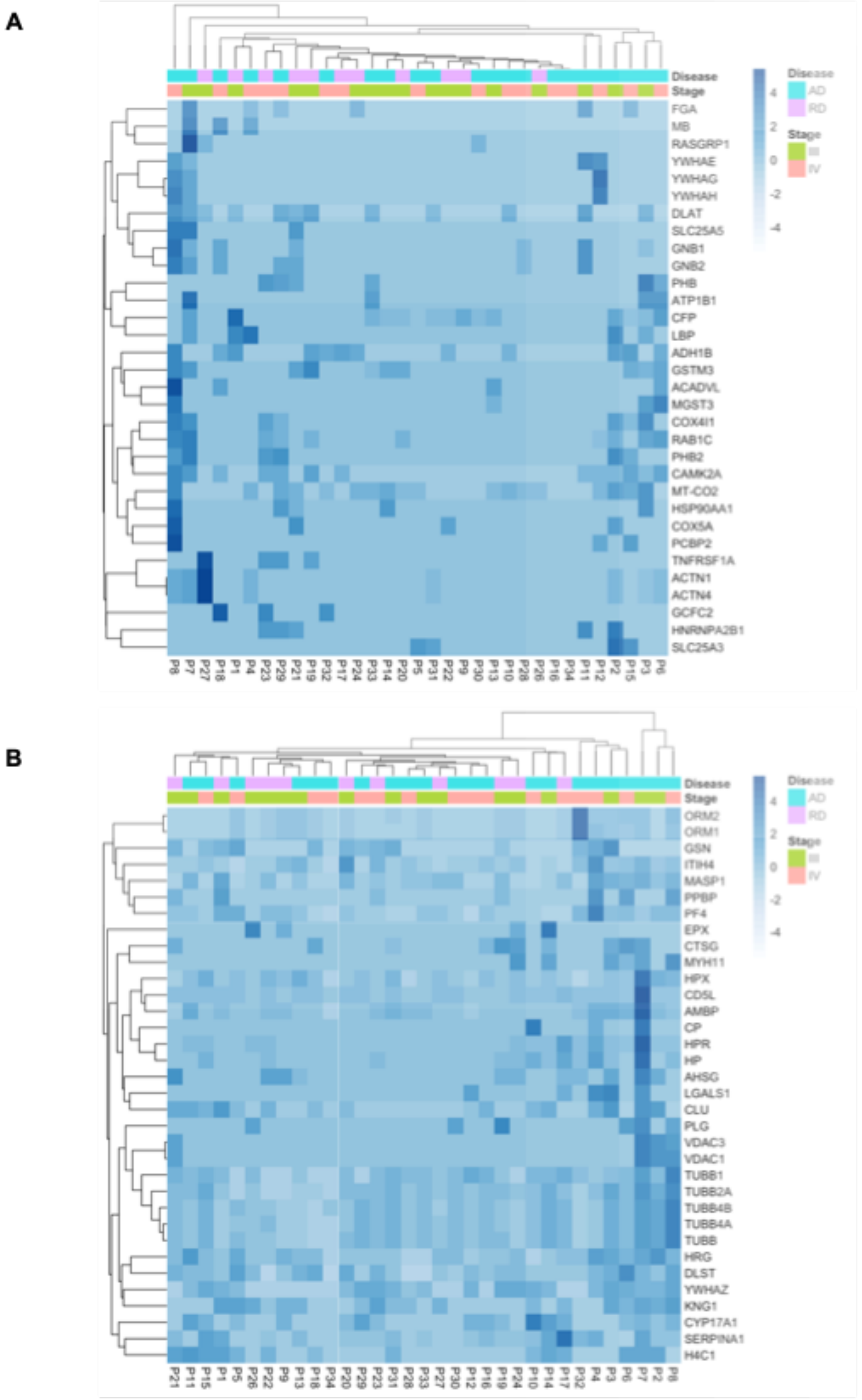

**Supplementary Figure 11: Heatmap of serum immuno-mass spectrometry (IMS) peak area.** Heatmap of immuno-mass spectrometry (IMS) peak area annotated with information on stage (III or IV) and active (AD) or resected (RD) disease status, for autoantibodies found in melanoma patients only (A) and in both melanoma patients and healthy volunteers (B) serum.

**Supplementary Figure 12**

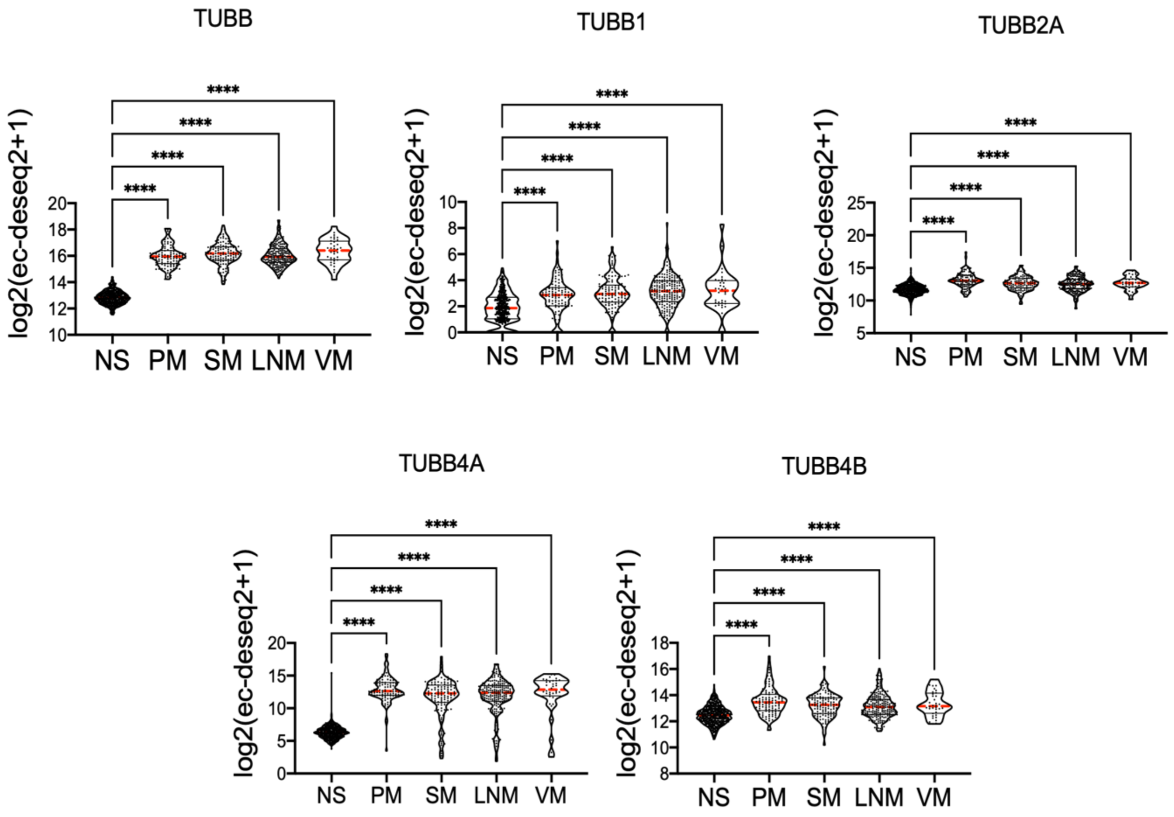


**Supplementary Figure 12: Gene expression of tubulins in human skin and melanoma lesions.** Gene expression of TUBB, TUBB1, TUBB2A, TUBB4A, TUBB4B in: normal skin, NS (n = 555); primary melanoma, PM (n = 102); melanoma skin metastases, SM (n = 116); lymph node metastases, LNM (n = 208); visceral metastases, VM (n = 36); (RSEM expected count (DESeq2 standardized) dataset, TCGA TARGET GTEx study, UCSC Xena). Statistical significance was calculated with non parametric ANOVA (Kruskal-Wallis test); *P < 0.05, **P < 0.01, ***P < 0.001 and ****P < 0.0001.

## Supplementary Figure 13

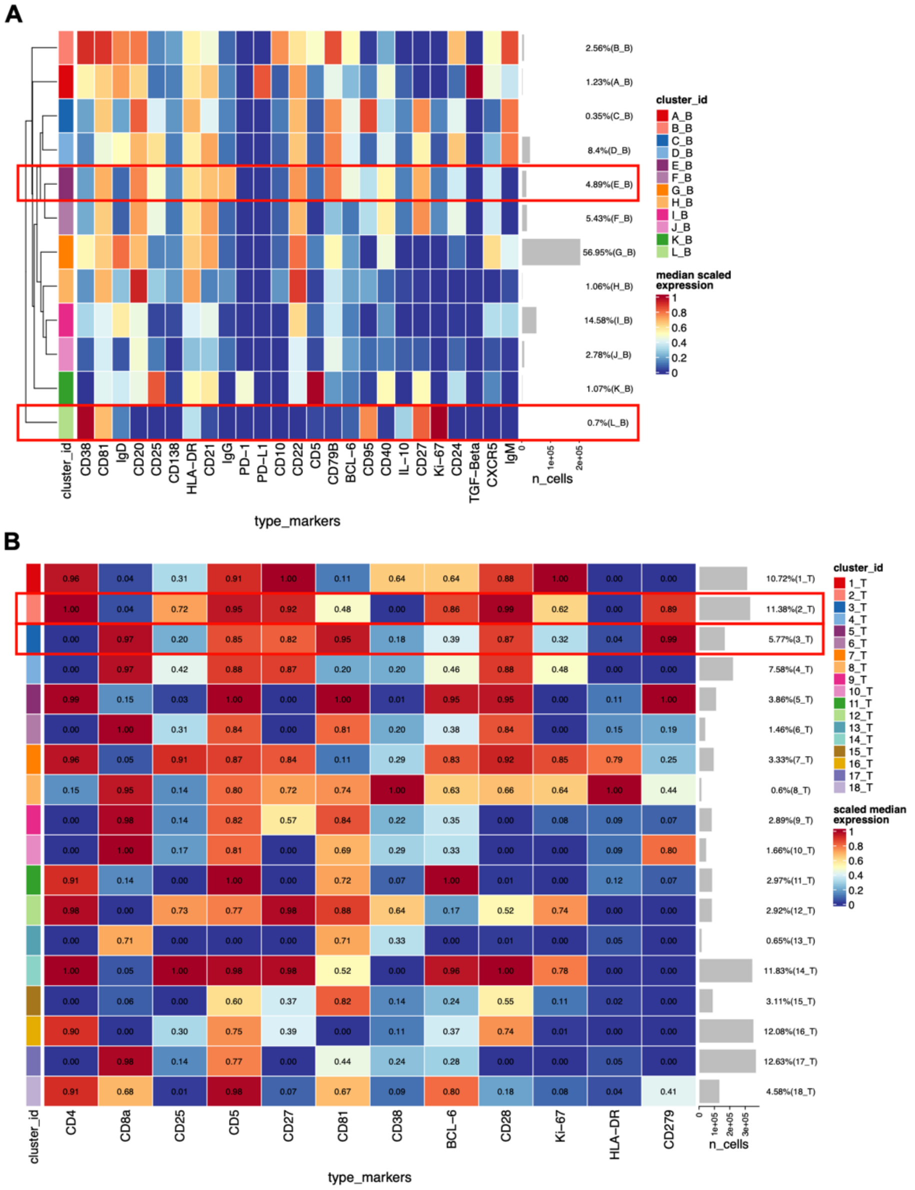


**Supplementary Figure 13: Heatmaps of marker expression for B cells and T cells detected by CyTOF analyses in melanoma patient blood samples.** Heatmaps represent the median scaled expression of the markers used for B cell (**A**) or T cell (**B**) clustering. Red rectangles indicate the clusters of interest.

**Supplementary Table 1: Criteria used for labelling and merging CyTOF clusters [2].**

| Cluster | Markers | Population |
|---|---|---|
| A | CD27- IgD++ IgMlow CD38+ CD24- TGFβ + PDL1+ | Naïve TGFβ+ PDL1+ |
| B | CD27+ IgD- IgM++ CD38low CD24+ | IgM+ memory |
| C | CD27+ IgD+ IgM++ CD38+/- CD24+ | IgD+ IgM+ memory |
| D | CD27+ IgD+ IgM++ CD38+/- CD24- CD21- CD95- BCL6+ | IgD+ IgM+ memory |
| E | CD27+ IgD- IgM- IgGlow CD38+/- CD24+ CD21+ CD95+/- | Class switched memory (resting) |
| F | CD27- IgD- IgM- IgG++ CD38+ CD24low CD21+ CXCR5+ | Double negative (DN1) |
| G | CD27+ IgD- IgM- IgG- CD38+/- CD24- CD21+ CD95++ CD79Blow | Class switched memory (activated) |
| H | CD27- IgD+ IgMlow CD38+ CD24- CD21+ CD95- | Naïve |
| J | CD27- IgD++ IgM++ CD38++ CD24+ CD10+ | Transitional |
| K | CD27- IgD- IgM- IgG++ CD38+/- CD24- CD21- CXCR5- | Double negative (DN2) |
| L | CD27- IgM+/- IgD++ CD24- CD38- CD21- | Naïve (activated) |
| M | CD27+/- IgM+ IgD+/- CD24+/- CD38- | Naïve |
| N | CD27- IgM+/- IgD+ CD24- CD38+/- CXCR5+ | Naïve |
| O | CD27- IgM- IgD- CD24- CD38+/- CD79- | Double negative (CD79b-) |
| P | CD27+ IgG- IgM- IgD+/- CD24+/- CD38- CD21+ CD5+++ CD25+++ PD1+ CD79B- | IgDlow memory |
| Q | CD27+++ IgG- IgM- IgD- CD24- CD38+++ CD95++ ki67+++ CD79B- | Plasmablasts |

**Supplementary Table 2: Number of sequences per melanoma skin metastasis sample derived in high throughput antibody repertoire analyses.**

| Donor | Number of total sequences | | | |
|---|---|---|---|---|
| | IgH | IgK | IgL | Number of IgH clones |
| P55 | 21,312 | 4,929 | 5,404 | 82 |
| P58 | 87,565 | 83,417 | 86,190 | 1263 |
| P66 | 67,144 | 78,023 | 77,111 | 443 |
| P71 | 5,605 | 5,204 | 6,708 | 104 |
| P84 | 43,389 | 13,558 | 14,220 | 994 |

**Supplementary Table 3: Number of heavy chain sequences derived per cohort in high throughput antibody repertoire.**

| Cohort | Number of samples | Number of unique sequences |
|---|---|---|
| HV (Healthy Volunteer) | 9 | 68,347 |
| MP (Melanoma Patient) | 5 | 27,506 |
| EB (ebola) | 12 | 60,020 |
| Covid-19 | 16 | 331,159 |
| Tonsils | 8 | 1,429,393 |

**Supplementary Table 4: Melanoma patients recruited for PBMC analysis by CyTOF.**

| Sample code | Gender | Age | Stage at sampling |
|---|---|---|---|
| P2 | M | 85 | IIIC |
| P3 | M | 88 | IIIC |
| P4 | M | 78 | IV |
| P7 | M | 86 | IIIC |
| P8 | F | 75 | IV |
| P10 | F | 78 | IV |
| P11 | M | 79 | IIIB |
| P12 | F | 79 | IV |
| P13 | F | 64 | IIIC |
| P14 | F | 82 | IIID |
| P15 | M | 77 | IV |
| P16 | M | 80 | IV |
| P18 | M | 73 | IV |
| P23 | F | 77 | IV |
| P24 | F | 64 | III |
| P26 | F | 69 | IIIC |
| P27 | M | 60 | IIIC |
| P32 | M | 67 | IV |
| P33 | M | 57 | IIIC |
| P35 | F | 76 | IIIB |
| P36 | M | 81 | IV |
| P37 | M | 85 | IV |
| P38 | F | 65 | III |
| P39 | M | 72 | IIIC |
| P40 | F | 85 | IV |
| P41 | F | 79 | IIIC |
| P42 | M | 77 | IV |
| P43 | M | 61 | IIIC |
| P44 | F | 65 | IV |
| P45 | F | 80 | IIIC |
| P46 | M | 71 | IV |
| P47 | F | 68 | IV |
| P48 | F | 68 | IV |
| P49 | M | 74 | III |
| P50 | F | 83 | IIIC |

**Supplementary Table 5: Healthy volunteers recruited for PBMC analysis by CyTOF.**

| **Sample code** | **Gender** | **Age** |
|---|---|---|
| HV1 | F | 59 |
| HV2 | F | 72 |
| HV3 | F | 68 |
| HV4 | M | 35 |
| HV5 | M | 76 |
| HV6 | F | 77 |
| HV7 | F | 72 |
| HV8 | F | 55 |
| HV9 | F | 61 |
| HV10 | F | 59 |
| HV11 | F | 88 |
| HV12 | F | 37 |
| HV13 | M | 28 |

**Supplementary Table 6: Melanoma patients recruited for PBMC analysis by Flow Cytometry.**

| Sample code | Gender | Age | Stage at sampling |
|---|---|---|---|
| P51 | M | 56 | IV |
| P52 | F | 44 | IIIC |
| P53 | F | 77 | IIIA |
| P54 | M | 41 | IV |
| P55 | M | 66 | IV |
| P56 | M | 45 | IV |
| P57 | M | 47 | IV |
| P58 | F | 67 | IIIC |
| P59 | M | 48 | IV |
| P60 | M | 65 | IV |
| P61 | M | 60 | IV |
| P62 | F | 81 | IV |
| P63 | M | 47 | IIIC |
| P64 | F | 59 | IV |
| P65 | F | 84 | III |
| P66 | M | 80 | IIIB |
| P67 | M | 70 | IV |
| P68 | M | 31 | IV |
| P69 | F | 71 | IV |
| P70 | M | 59 | IIIC |
| P71 | F | 60 | IV |
| P72 | F | 80 | IIB |
| P73 | F | 77 | IIIB |
| P74 | M | 74 | IIIC |
| P75 | M | 89 | IV |
| P76 | F | 80 | IV |
| P77 | F | 83 | IIIC |
| P78 | M | 95 | IIIB |
| P79 | M | 77 | IIIC |

**Supplementary Table 7: Melanoma lesion specimens.**

| Sample code | Age | Gender | Stage | Lesion type | Reason for exclusion from the analysis | TILs* in original primary melanoma |
|---|---|---|---|---|---|---|
| P55-1 | 53 | F | IIIB | Subcutaneous metastasis | | Present: non-brisk |
| P55-2 | 54 | F | IIIB | In transit melanoma | | Present: non-brisk |
| P56 | 45 | M | IV | Cutaneous primary lesion | | N/A |
| P57 | 61 | F | IIIC | Cutaneous metastasis | | Absent |
| P58 | 76 | F | IIC | Subcutaneous in-transit metastasis | | Absent |
| P59 | 49 | M | IIB | Desmoplastic melanoma, primary lesion | | Present: non brisk from desmoplastic lesion |
| P66 | 76 | M | IV | Subcutaneous metastasis | | Present: non-brisk |
| P71 | 70 | M | IV | Subcutaneous metastasis | | Present: non-brisk |
| P77 | 80 | F | IIB | Subcutaneous in-transit metastasis | less than 10 B cells | N/A |
| P79 | 74 | M | IIIC | Subcutaneous in-transit metastasis | less than 10 B cells | Present: non-brisk |
| P80 | 80 | F | IV | Subcutaneous in-transit metastasis | | Present: non-brisk |
| P81 | 83 | F | IIIC | Subcutaneous in-transit metastasis | less than 10 B cells | Absent |
| P82 | 95 | M | IIIB | Subcutaneous in-transit metastasis | | Present: non-brisk |
| P83 | 77 | M | IIIC | Subcutaneous in-transit metastasis | less than 10 B cells | Present: non-brisk |
| P84 | 66 | F | IIA | Cutaneous metastasis | | Present: non-brisk |
| P85 | M | F | IB | Primary cutaneous melanoma | No TILs in sample | N/A |
| P86 | 70 | F | IV | Recurrent subcutaneous metastasis | less than 10 B cells | Present: non-brisk |
| P87 | 84 | M | IV | Subcutaneous in-transit metastasis | No memory B cells in TILs | Present: non-brisk |

* TILs: tumor infiltrating lymphocytes

**Supplementary Table 8: Melanoma patients recruited for serum immuno-mass spectrometry analysis.**

| Sample code | Gender | Age | Stage at sampling | Autoimmune related co-morbidities |
|---|---|---|---|---|
| P1 | M | 57 | IIIB | |
| P2 | M | 85 | IIIC | Ulcerative colitis |
| P3 | M | 88 | IIIC | |
| P4 | M | 78 | IV | |
| P5 | F | 35 | IV | |
| P6 | F | 35 | IV | |
| P7 | M | 86 | IIIC | |
| P8 | F | 75 | IV | |
| P9 | M | 58 | IIIC | |
| P10 | F | 78 | IV | |
| P11 | M | 79 | IIIB | |
| P12 | F | 79 | IV | |
| P13 | F | 64 | IIIC | |
| P14 | F | 82 | IIID | |
| P15 | M | 77 | IV | Baseline autoimmune thyroid disease |
| P16 | M | 80 | IV | |
| P17 | M | 57 | IV | |
| P18 | M | 73 | IV | |
| P19 | M | 83 | IIIC | |
| P20 | F | 72 | IIIC | |
| P21 | M | 49 | IIIB | |
| P22 | M | 44 | IIIA | |
| P23 | F | 77 | IV | |
| P24 | F | 64 | III | |
| P26 | F | 60 | IIIC | Baseline autoimmune thyroid disease, polymyalgia rheumatica |
| P27 | F | 69 | IIIC | |
| P28 | M | 60 | IIIC | |
| P29 | M | 60 | IV | |
| P30 | F | 65 | IV | |
| P31 | M | 83 | IV | |
| P32 | F | 80 | IIIC | |
| P33 | M | 67 | IV | |
| P34 | M | 57 | IIIC | |

**Supplementary Table 9: CyTOF antibody panel.**

| Metal Tag | Antibody | Clone | Source |
|---|---|---|---|
| 89Y | CD45 | HI30 | Fluidigm |
| 141Pr | CD3 | UCHT1 | Fluidigm |
| 142Nd | CD19 | HIB19 | Fluidigm |
| 144Nd | CD38 | HIT2 | Fluidigm |
| 145Nd | CD81 | 5A6 | Fluidigm |
| 146Nd | IgD | IA6-2 | Fluidigm |
| 147Sm | CD20 | 2H7 | Fluidigm |
| 148Nd | CD8a | SK1 (Custom) | Biolegend |
| 149Sm | CD25 (IL2R) | 2A3 | Fluidigm |
| 150Nd | CD138 | DL-101 | Fluidigm |
| 151Eu | HLA-DR | G46-6 | Fluidigm |
| 152Sm | CD21 | BL13 | Fluidigm |
| 153Eu | Ig Lambda | MHL-38 (Custom) | Biolegend |
| 154Sm | IgG R10 | R10 (Custom) | RND |
| 155Gd | CD279 (PD-1) | EH12.2H7 | Fluidigm |
| 156Gd | CD274 (PD-L1) | 29E.2A3 | Fluidigm |
| 158Gd | CD10 | HI10a | Fluidigm |
| 159Tb | CD22 | HIB22 | Fluidigm |
| 160Gd | Ig Kappa | MHK-49 | Fluidigm |
| 161Dy | CD5 | UCHT2 (Custom) | Biolegend |
| 162Dy | CD79B | CB3-1 | Fluidigm |
| 163Dy | BCL-6 | K112-91 | Fluidigm |
| 164Dy | CD95/Fas | DX2 | Fluidigm |
| 165Ho | CD40 | 5C3 | Fluidigm |
| 166Er | IL-10 | JES3-9D7 | Fluidigm |
| 167Er | CD27 | L128 | Fluidigm |
| 168Er | Ki-67 | B56 | Fluidigm |
| 169Tm | CD24 | ML5 | Fluidigm |
| 170Er | TGF-Beta | TW4-6H10 (Custom) | Biolegend |
| 171Yb | CD185 (CXCR5) | 51505 | Fluidigm |
| 172Yb | IgM | MHM-88 | Fluidigm |
| 175Lu | CD28 | CD28.2 (Custom) | Biolegend |
| 176Yb | CD4 | RPA-T4 | Fluidigm |
| 209Bi | CD16 | 3G8 | Fluidigm |

**Supplementary Methods**

**Immuno-mass spectrometry**

*Tissue protein extraction*: Fresh frozen skin (n = 3) and melanoma (n = 2) tissue sections collected at autopsy or surgery, respectively. Samples were cut and weighed (~500-700 mg wet weight). Tissues were individually pulverized in liquid nitrogen with a mortar and pestle to yield a fine powder. Proteins were extracted and solubilized using 0.2% RapiGest (Waters Corporation, Milford, USA) in 50 mM ammonium bicarbonate (ABC). The pulverized tissue samples were vortexed every 5 minutes on ice, for 30 minutes, and then sonicated on ice three times for 15 s with the MISONIX immersion tip sonicator (Q SONICA LLC, CT, USA). The tissue samples were centrifuged at 15,000 x g at 4°C for 20 minutes and the supernatant was collected. A Pierce BCA Protein Assay (Thermo Fisher Scientific, San Jose, California) was performed on each tissue protein extract for total protein quantification. Melanoma tissue lysates were pooled in equal parts (in terms of total protein contribution).

*Monoclonal IgG antibody immunoprecipitation:* Unknown IgG monoclonal antibodies (n = 12) were purified using protein G magnetic beads (Protein G Mag Sepharose Xtra, GE Healthcare, Chicago, USA). Briefly, 50 µl of 10% w/v medium slurry was resuspended and added to each microcentrifuge tube. The storage solution was removed using a magnetic particle concentrator (MPC-E, Dynal, Oslo, Norway). The magnetic beads were then equilibrated with 500 µl of 1X PBS. Following the removal of the 1X PBS solution, 50 µl of each unknown IgG monoclonal antibody (1 µg/µl) was diluted to a final volume of 300 µl using 1X PBS and added to the magnetic beads. CSPG4 and NIP monoclonal antibodies served as a positive and negative control, respectively and were processed separately from the unknown IgG monoclonal antibodies. Samples were incubated for 30-minutes with gentle rotation. The magnetic beads were then washed twice with 500 µl 1X PBS and incubated with 100 µg of pooled melanoma

tissue lysate for 2 hours with gentle rotation. The magnetic beads were then washed two times with 500 µl 1X PBS 0.05% Tween 20. This was followed by an additional two washes with 500 µl 1X PBS. The beads were resuspended in 100 µl of PBS for on-bead trypsin digestion. The immuno-captured monoclonal antibodies and their cognate target antigens were reduced with 100 mM DTT added to a final concentration of 5 mM and incubated for 40 minutes at 56°C. The samples were then alkylated with 500 mM IAA added to a final concentration of 15 mM and incubated for 30 minutes at room temperature, in the dark and with constant shaking. Trypsin was added in a 1:50 ratio and the samples were incubated overnight (18 hours) at 37 °C with constant shaking. The next day, samples were added to the magnetic apparatus. The supernatant was removed and acidified with ~1% formic acid (pH 2) to inactivate trypsin. All monoclonal antibodies were processed in duplicate, including the CSPG4 positive control. The NIP negative control was processed in triplicate.

*Liquid chromatography coupled to tandem mass spectrometry (LC-MS/MS):* In all samples, peptides were extracted from solution using C18 OMIX tips (Agilent Technologies, Santa Clara, CA) and eluted in 3 µl of elution buffer B (65% acetonitrile, 0.1% formic acid). Fifty-seven µl of buffer A (0.1% formic acid) were added to each sample and 18 µl of sample was loaded from a 96-well microplate autosampler using the EASY-nLC 1000 system (Thermo Fisher Scientific) onto a 75 µm x 3.3 cm capillary (IntegraFrit Capillary, New Objective, Woburn, Massachusetts) packed-in-house with a C18 Agilent Pursuit 5 µm bead media (Agilent Technologies, Santa Clara, California). Peptides were eluted from the trap column at 300 nl/min with an increasing concentration of Buffer D (0.1% formic acid in 100% acetonitrile) over a 30 min gradient onto a resolving 15 cm x 75 µm ID analytical column (PicoFrit Capillary, New Objective) packed-in-house with C18 Agilent Pursuit 3 µm bead media (Agilent Technologies). The liquid chromatography setup was coupled online to Q Exactive H-FX (Thermo Fisher Scientific) mass spectrometer using the NanoSpray Flex

ionization source (Thermo Fisher Scientific) with the capillary temperature set to 320 °C and a spray voltage of 2kV. A 60 min data-dependent acquisition (DDA) method performed a full MS1 scan from 400–1500 m/z at a resolution of 70,000 in profile mode. This was followed by fragmentation of the top 12 parent ions using the HCD cell and detection of fragment ions at a resolution of 17,500. The following MS method parameters were used: MS1 Automatic Gain Control (AGC) target was set to 1e6 with maximum injection time (IT) of 50 ms; MS2 AGC was set to 5e4 with maximum IT of 110 ms; isolation window was 1.6 m/z; intensity threshold was 9.1e3; normalized collision energy (NCE) was set to 27; charge exclusion was set to fragment only 2+,3+, 4+ charge state ions; peptide match was set to preferred and dynamic exclusion was set to 18 s. Blank injections of buffer A (1 µl) were run between duplicates and reproducibility was confirmed by running 0.1 fmol/µl bovine serum albumin (BSA), every 10 runs.

*Parallel reaction monitoring mass spectrometry (PRM-MS):* Additionally, peptides extracted for LC-MS/MS analysis were analyzed using a targeted parallel reaction monitoring (PRM) MS approach. Samples were loaded (18 µl) onto a 3.2 cm C18 trap column (5 µm C18 particle, 150 µm inner diameter) using the using the EASY-nLC 1000 system (Thermo Fisher Scientific) running buffer A. Peptides were eluted from the trap column at 300 nl/min onto a resolving 15 cm long PicoTip Emitter (3 µm C18 particle) with an inner diameter of 75 µm (8 µm tip, New Objective). The LC setup was coupled online to a Q-Exactive Plus (Thermo Fisher Scientific) mass spectrometer with a nanoelectrospray ionization source. A 60 min DDA method was setup on the Q-Exactive Plus. The full MS1 scan from 400 to 1,500 m/z was acquired in the Orbitrap at a resolution of 70,000. AGC for MS1 was set to $1 \times 10^6$ with a maximum injection time of 120 ms. AGC target for MS2 was set to $2 \times 10^5$ with a maximum injection time of 130 ms, normalized collision energy (NCE) was set to 27. Reproducibility was confirmed by running a quality control solution of 0.1 fmol/µl BSA.

*Data analysis:* XCalibur software v.2.0.6 (Thermo Fisher Scientific) was used to generate raw files. Raw files were uploaded into Proteome Discoverer v. 1.4 (Thermo Fisher Scientific) and searched with Sequest HT search engine against the Human 5640 Swiss-Prot protein database (January 2018). Search parameters included trypsin (full) enzyme digestion, a maximum of two missed cleavages, static modification of cysteine carbamidomethylation, dynamic modification of methionine oxidation, precursor mass tolerance of 7 ppm, fragment mass tolerance of 0.02 Da, 1% false-discovery rate (FDR) at the peptide and protein level using the Percolator node.

## Supplementary References

1. Bashford-Rogers RJM, Smith KGC, Thomas DC. Antibody repertoire analysis in polygenic autoimmune diseases. Immunology. 2018 Sep;155(1):3-17. doi: 10.1111/imm.12927. Epub 2018 Apr 16. PMID: 29574826; PMCID: PMC6099162.

2. Sanz I, Wei C, Jenks SA, Cashman KS, Tipton C, Woodruff MC, Hom J, Lee FE. Challenges and Opportunities for Consistent Classification of Human B Cell and Plasma Cell Populations. Front Immunol. 2019 Oct 18;10:2458. doi: 10.3389/fimmu.2019.02458
