# Supplementary material for: B cell profiles, antibody repertoire and reactivity reveal dysregulated responses with autoimmune features in melanoma
Source: Nat Commun. 2023 Jun 8;14:3378. doi: 10.1038/s41467-023-39042-y (PMC10249578; doi:10.1038/s41467-023-39042-y)
Supplement: Supplementary file 2 — Reporting Summary [file 41467_2023_39042_MOESM2_ESM.pdf]

nature portfolio

Corresponding author(s): Prof Sophia Karagiannis (NCOMMS-22-21570)

Last updated by author(s): Apr 21, 2023

Reporting Summary

Nature Portfolio wishes to improve the reproducibility of the work that we publish. This form provides structure for consistency and transparency in reporting. For further information on Nature Portfolio policies, see our Editorial Policies and the Editorial Policy Checklist.

Statistics

For all statistical analyses, confirm that the following items are present in the figure legend, table legend, main text, or Methods section.

n/a	Confirmed	
☐	☒	The exact sample size (*n*) for each experimental group/condition, given as a discrete number and unit of measurement
☐	☒	A statement on whether measurements were taken from distinct samples or whether the same sample was measured repeatedly
☐	☒	The statistical test(s) used AND whether they are one- or two-sided<br>*Only common tests should be described solely by name; describe more complex techniques in the Methods section.*
☒	☐	A description of all covariates tested
☐	☒	A description of any assumptions or corrections, such as tests of normality and adjustment for multiple comparisons
☐	☒	A full description of the statistical parameters including central tendency (e.g. means) or other basic estimates (e.g. regression coefficient) AND variation (e.g. standard deviation) or associated estimates of uncertainty (e.g. confidence intervals)
☐	☒	For null hypothesis testing, the test statistic (e.g. *F*, *t*, *r*) with confidence intervals, effect sizes, degrees of freedom and *P* value noted<br>*Give P values as exact values whenever suitable.*
☐	☒	For Bayesian analysis, information on the choice of priors and Markov chain Monte Carlo settings
☒	☐	For hierarchical and complex designs, identification of the appropriate level for tests and full reporting of outcomes
☒	☐	Estimates of effect sizes (e.g. Cohen's *d*, Pearson's *r*), indicating how they were calculated

Our web collection on statistics for biologists contains articles on many of the points above.

Software and code

Policy information about availability of computer code

Data collection	Mass cytometry data were acquired using CyTOF® Software v7.0.8493 for Fluidigm Helios. Flow cytometry data were acquired using BD FACSDiva v6.0 software. The publicly available dataset GSE123139 was acquired using R (RStudio, Version 1.3.1093). RSEM expected count (DESeq2 standardized) dataset from TCGA TARGET GTEx study (UCSC Xena) were obtained using Xena Browser (https://xenabrowser.net). Immunoglobulin long read sequences were acquired using PacBio Sequel 2 technology. Immuno mass spectrometry row data were generated using XCalibur software v.2.0.6 (Thermo Fisher Scientific). Immunofluorescence images were acquired with NIS-Elements software (Nikon).
Data analysis	Flow and mass cytometry data were analyzed with FlowJo v10. Statistical analyses were preformed with GraphPad Prism v9. B cell phenotyping was performed using R (RStudio, Version 1.3.1093): mass cytometry data were analyzed using a modified R script based on the CATALYST, diffCYT, FlowSOM, edgeR and flowCORE packages which can be found using the bioconductor terminal https://www.bioconductor.org/packages/release/bioc/vignettes/CATALYST/inst/doc/differential.html; single cell RNAseq analysis was performed using the Seurat package (4.0.6); B cell repertoire analyses were performed using IMGT/HighV-QUEST (Brochet et al., 2008, Giudicelli et al., 2011), BWA-MEM algorithm (version 0.7.17.1 on Galaxy [https://usegalaxy.org]) (https://arxiv.org/abs/1303.3997), BRepertoire webserver, BrepPhylo (https://github.com/Fraternalilab/BrepPhylo) using the PHYLIP algorithm and the ETE Toolkit 3 in the R statistical programming environment (v3.6.2). Immuno mass spectrometry row files were uploaded into Proteome Discoverer v. 1.4 (Thermo Fisher Scientific) and searched with Sequest HT search engine against the Human 5640 Swiss-Prot protein database, peptides peak area analyses were performed with R (RStudio, Version 1.3.1093) Rvenn package and GraphPad Prism v9. Immunofluorescence images were analyzed with ImageJ Fiji (v2) and QuPath (v0.2.3).

For manuscripts utilizing custom algorithms or software that are central to the research but not yet described in published literature, software must be made available to editors and reviewers. We strongly encourage code deposition in a community repository (e.g. GitHub). See the Nature Portfolio guidelines for submitting code & software for further information.

Data

Policy information about availability of data

All manuscripts must include a data availability statement. This statement should provide the following information, where applicable:

- Accession codes, unique identifiers, or web links for publicly available datasets
- A description of any restrictions on data availability
- For clinical datasets or third party data, please ensure that the statement adheres to our policy

The data generated in this study are provided in the Supplementary Information/Source Data file. The computer code generated in this study has been deposited in the following github repository: https://github.com/josef0731/melanoma-ig.

Human research participants

Policy information about studies involving human research participants and Sex and Gender in Research.

Reporting on sex and gender	Information on patients' and healthy volunteers' sex was collected based on self-reporting. Patients group has been designed to be sex-homogeneous and sex-based analyses were not included in this study.
Population characteristics	We selected metastatic melanoma patients (stage III and IV) who had not received immunotherapy treatment. Patients were male and female over the age of 18 years. We selected healthy volunteers with no history of malignancy to match patients' age.
Recruitment	This study focus on immunotherapy treatment naive patients (male and female) with metastatic melanoma (stages III - IV) over the age of 18 years and able to provide informed written consent were included and healthy volunteers (male and female) over the age of 18 years with no history of malignancy and able to provide informed written consent were included. Age might influence the distribution of B cell populations, for this reason healthy volunteers were selected to match patients' age.<br>Participants were identified independently by a study manager at the melanoma multidisciplinary team meeting and via the melanoma outpatient clinic at St John's Institute of Dermatology, Guy's and St Thomas' NHS Trust (GSTT). Healthy volunteer blood donation calls were placed on the King's College London and GSTT intranet in addition to the King's College London research circular email. All human blood and tumor samples were collected with written informed consent.
Ethics oversight	The study was conducted at King's College London, Guy's and St Thomas' NHS Foundation Trust (REC reference: 08/H0804/139 approved by London Bridge NRES committee; REC reference: 16/LO/0366 approved by London-Central NRES Committee).

Note that full information on the approval of the study protocol must also be provided in the manuscript.

Field-specific reporting

Please select the one below that is the best fit for your research. If you are not sure, read the appropriate sections before making your selection.

☒ Life sciences ☐ Behavioural & social sciences ☐ Ecological, evolutionary & environmental sciences

For a reference copy of the document with all sections, see nature.com/documents/nr-reporting-summary-flat.pdf

Life sciences study design

All studies must disclose on these points even when the disclosure is negative.

Sample size	For CyTOF, flow cytometry and immuno-mass spectrometry analyses we have chosen a sample size of at least 25 patients (35, 29 and 33 patients, respectively), and we selected 13 age matched healthy volunteers for CyTOF analyses. Due to the variability of the parameters object of our study in tumor patients we chose to recruit a higher number of patients compared to healthy volunteers. The tumor samples analyzed by flow cytometry were 17, while, for tissue size availability and complexity of the experiment reasons, for spatial transcriptomics and Ig repertoire analyses we chose a sample size of 5 patients.
Data exclusions	To characterize the B cell phenotypes in blood and tumor samples, of the 17 tumor samples analyzed by flow cytometry, 7 samples were excluded from the analysis because of the lack of B cells in the tumor (less than 10 B cells detected).
Replication	In this study we characterize B cell phenotypes, antibody repertoire and serum antibodies reactivity in melanoma patients. Each sample was considered biologically independent. Furthermore, when the samples have been analyzed in batches, we confirmed there were no batch effects.
Randomization	Experimental groups were determined based on demographic information: we compared melanoma patients versus healthy volunteers, or melanoma patients' blood versus tumor, or samples from healthy volunteers versus stage III and stage IV patients.

Blinding

The aim of this study is to characterize melanoma patients' B cell phenotypes, antibody repertoire and serum antibodies reactivity in immunotherapy naive patients, without the aim of correlating it to treatment outcome, for these reasons blinding was not relevant to this study.

Reporting for specific materials, systems and methods

We require information from authors about some types of materials, experimental systems and methods used in many studies. Here, indicate whether each material, system or method listed is relevant to your study. If you are not sure if a list item applies to your research, read the appropriate section before selecting a response.

Materials & experimental systems

n/a	Involved in the study	
☐	☒	Antibodies
☐	☒	Eukaryotic cell lines
☒	☐	Palaeontology and archaeology
☒	☐	Animals and other organisms
☒	☐	Clinical data
☒	☐	Dual use research of concern

Methods

n/a	Involved in the study	
☒	☐	ChIP-seq
☐	☒	Flow cytometry
☒	☐	MRI-based neuroimaging

Antibodies

Antibodies used

89Y CD45 clone HI30 (Fluidigm, 3089003B) 0.6 µL/sample
141Pr CD3 clone UCHT1 (Fluidigm 3141019B) 0.75 µL/sample
142Nd CD19 clone HIB19 (Fluidigm, 3142001B) 0.4 µL/sample
144Nd CD38 clone HIT2 (Fluidigm, 3144014B) 0.5 µL/sample
145Nd CD81 clone 5A6 (Fluidigm, 3145007B) 0.5 µL/sample
146Nd IgD clone IA6-2 (Fluidigm, 3146005B) 0.5 µL/sample
147Sm CD20 clone 2H7 (Fluidigm, 3147001B) 0.6 µL/sample
148Nd CD8a clone SK1 (Custom) (Biolegend, 344702) 0.75 µL/sample
149Sm CD25 (IL2R) clone 2A3 (Fluidigm, 3149010B) 0.7 µL/sample
150Nd CD138 clone DL-101 (Fluidigm, 3150012B) 0.75 µL/sample
151Eu HLA-DR clone G46-6 (Fluidigm, 3151023B) 0.25 µL/sample
152Sm CD21 clone BL13 (Fluidigm, 3152010B) 0.5 µL/sample
153Eu Ig Lambda clone MHL-38 (Custom) (Biolegend, 316602) 0.5 µL/sample
154Sm IgG clone 1268A (Custom) R&D, MAB11013 0.5 µL/sample
155Gd CD279 (PD-1) clone EH12.2H7 (Fluidigm, 3155009B) 0.75 µL/sample
156Gd CD274 (PD-L1) clone 29E.2A3 (Fluidigm, 3156026B) 0.75 µL/sample
158Gd CD10 clone HI10a (Fluidigm, 3158011B) 0.7 µL/sample
159Tb CD22 clone HIB22 (Fluidigm, 3159005B) 0.5 µL/sample
160Gd Ig Kappa clone MHK-49 (Fluidigm, 3160005B) 0.5 µL/sample
161Dy CD5 clone UCHT2 (Custom) (Biolegend, 300602) 0.5 µL/sample
162Dy CD79B clone CB3-1 (Fluidigm, 3162008B) 0.5 µL/sample
163Dy BCL-6 clone K112-91 (Fluidigm, 3163012B) 0.5 µL/sample
164Dy CD95/Fas clone DX2 (Fluidigm, 3164008B) 0.5 µL/sample
165Ho CD40 clone 5C3 (Fluidigm, 3165005B) 0.5 µL/sample
166Er IL-10 clone JES3-9D7 (Fluidigm, 3166008B) 1 µL/sample
167Er CD27 clone L128 (Fluidigm, 3167006B) 0.4 µL/sample
168Er Ki-67 clone B56 (Fluidigm, 3168007B) 0.6 µL/sample
169Tm CD24 clone ML5 (Fluidigm 3169004B) 0.5 µL/sample
170Er TGF-Beta clone TW4-6H10 (Custom) (Biolegend, 349702) 1 µL/sample
171Yb CD185 (CXCR5) clone 51505 (Fluidigm, 3171014B) 0.75 µL/sample
172Yb IgM clone MHM-88 (Fluidigm, 3172004B) 0.3 µL/sample
175Lu CD28 clone CD28.2 (Custom) (Biolegend, 302902) 0.5 µL/sample
176Yb CD4 clone RPA-T4 (Fluidigm, 3176010B) 0.5 µL/sample
209Bi CD16 clone 3G8 (Fluidigm, 3209002B) 0.25 µL/sample
Anti CD45 PerCP (Biolegend, 304026) 5 µL/sample
Anti CD19 FITC (BD, 555412) 20 µL/sample
Anti CD27 BV421 (BD, 562513) 5 µL/sample
Anti IgD APC H7 (BD, 612798) 5 µL/sample
Anti IgM PECy7 (Biolegend, 314532) 5 µL/sample
rabbit anti-CD20 (clone EP459Y, abcam, ab78237) 1:100
mouse anti-Melanoma antibody mix (clones HMB45 + M2-7C10 + M2-9E3, abcam, ab732) 1:100

Validation

All antibodies were validated by the manufacturer: abcam, BD, Biolegend, R&D, Fluidigm. For the CyTOF panel, the antibodies that were readily bought from Fluidigm Inc. had undergone specific biological validation such that each reagent is detected and quantified with cytometry by time-of-flight mass spectrometry in the CyTOF system. The high purity and choice of metal isotopes ensure minimal background noise from signal overlap or endogenous cellular components. For the antibodies that were custom conjugated, technical advice was sought from the fluidigm field application specialists and conjugation was carried out using the Fluidigm metal-

labelling kits. Individual titrations were carried out on healthy controls and run as per the fluidigm validation protocol on the CyTOF system as detailed above.

Eukaryotic cell lines

Policy information about cell lines and Sex and Gender in Research

Cell line source(s)	Expi293F™ Cells are human cells derived from the 293F cell line (GIBCO brand cells).
Authentication	These cells were authenticated as being the core component of the Expi293 Expression System.
Mycoplasma contamination	The cells were regularly tested for mycoplasma contamination and we confirm that they were negative for mycoplasma.
Commonly misidentified lines (See ICLAC register)	NA

Flow Cytometry

Plots

Confirm that:

☒ The axis labels state the marker and fluorochrome used (e.g. CD4-FITC).

☒ The axis scales are clearly visible. Include numbers along axes only for bottom left plot of group (a 'group' is an analysis of identical markers).

☒ All plots are contour plots with outliers or pseudocolor plots.

☒ A numerical value for number of cells or percentage (with statistics) is provided.

Methodology

Sample preparation	Peripheral blood mononuclear cells (PBMCs) were isolated from 40 ml blood (or leukocyte cones) using Ficoll® Paque Plus density centrifugation (GE Healthcare). Melanoma and skin tissue were minced with a scalpel and then mechanically dissociated with gentleMACS dissociator in RPMI 1640 medium supplemented with 1 mM EDTA. The cell suspension and the remaining pieces of tissue were then incubated overnight at 37°C, 5% CO2 in RPMI 1640 medium supplemented with 10% heat inactivated Fetal Bovine Serum (FBS) and Penicillin-Streptomycin (10,000 U/ml) to allow the remaining immune cells to crawl out of the tissue. The cell suspension was then harvested, passed through a 100 µm cell strainer, and processed for B cell phenotyping. PBMC and tumor single cell suspensions were stained with LIVE/DEAD Fixable Aqua (Invitrogen) according to the manufacturer's instructions and then incubated with Fc Blocking Reagent (Miltenyi Biotec) for 10 min at room temperature followed by staining with anti-CD45 PerCP (BioLegend), anti-CD19 FITC (BD), anti-CD27 BV421 (BD) and anti IgD APC H7 (BD) (BioLegend) for 30 min at 4°C. The samples were then washed in Phosphate-Buffered Saline (PBS) 2% FBS (FACS Buffer), resuspended in 200 µl of FACS Buffer and analyzed by multicolor flow cytometry using BD FACSCanto II (BD Biosciences).
Instrument	FACS Canto II (BD)
Software	BD FACSDiva™ Software was used to acquire the data, FlowJo (v10) was used to analyze the data, and GraphPad Prism (v9) was used for statistical analysis.
Cell population abundance	We analysed B cells from Peripheral blood mononuclear cells (PBMCs), in blood B cells are around 10% of CD45+ cells. We single cell sorted IgD- CD27+ B cells directly into lysis buffer for RNA extraction and cDNA preparation so we could not check the purity after sorting.
Gating strategy	FSC and SSC parameters were used to exclude cell doublets and LIVE/DEAD Fixable Aqua was used to exclude dead cells. B cells were defined as CD45+ CD19+ cells. CD27 and IgD were used to define and characterize naive (CD27- IgD+) and memory (CD27+) B cells.

☒ Tick this box to confirm that a figure exemplifying the gating strategy is provided in the Supplementary Information.
